# Urban agglomeration worsens spatial disparities in climate adaptation

**DOI:** 10.1038/s41598-021-87739-1

**Published:** 2021-04-19

**Authors:** Seung Kyum Kim, Mia M. Bennett, Terry van Gevelt, Paul Joosse

**Affiliations:** 1 Faculty of Social Science, University of Hong Kong, 7.02C, The Jockey Club Tower, Centennial Campus, Pokfulam Road, Hong Kong SAR; 2 Department of Geography, University of Hong Kong, 10.37, The Jockey Club Tower, Centennial Campus, Pokfulam Road, Hong Kong SAR; 3 Department of Politics and Public Administration, University of Hong Kong, 9.48, The Jockey Club Tower, Centennial Campus, Pokfulam Road, Hong Kong SAR; 4 Department of Sociology, University of Hong Kong, 9.02, The Jockey Club Tower, Centennial Campus, Pokfulam Road, Hong Kong SAR

**Keywords:** Governance, Environmental health

## Abstract

Many countries promote urban agglomeration to enhance economic competitiveness, but the impacts of this strategy on local climate adaptation remain poorly understood. Here, we use variation in greenspaces to test the effectiveness of climate adaptation policy across climate impacts and vulnerability dimensions. Using satellite imagery and logistic regression, we analyze spatiotemporal correlation between greenspace and climate vulnerability in the Guangdong-Hong Kong-Macau Greater Bay Area, an area comprising ~ 70 million people and 11 cities, making it a useful natural experiment for our study. We find that while greenspace increases proportionally with climate exposure and sensitivity, many cities exhibit discrepancies between greenspace variation and climate vulnerability. Green adaptation funnels into wealthier, less vulnerable areas while bypassing more vulnerable ones, increasing their climate vulnerability and undermining the benefits of urban agglomeration. The results suggest that centrally-planned climate adaptation policy must accommodate local heterogeneity to improve urban sustainability. By neglecting local heterogeneity, urban agglomeration policy risks exacerbating spatial inequalities in climate adaptation.

## Introduction

Governments from municipal to national scales frequently promote urban agglomeration as a means of increasing cities' competitiveness within the global economy^1^. This strategy requires the effective integration of a complex set of institutional structures across a range of scales. While economic considerations dominate such efforts, given the rapid pace of climate change and increasing recognition of its economic effects^2,3^, there is an urgent need to develop effective climate adaptation and resilience-building strategies. Yet climate change affects communities and cities disproportionately across socioeconomic classes^4^. Urban integration based on prioritizing economic and institutional structures may hinder consideration of local variations in climate vulnerability in the decision-making process of local climate strategies, leading to greater spatial inequality in the provision of climate adaptation^5^.

Within agglomerating regions, the integration of policy across existing jurisdictional boundaries may lead to the disproportionate distribution of resources across cities with differing economic characteristics, health service capacities, and environmental attributes^6^. The push to integrate, particularly when it occurs in ways that ignore local environmental factors, can therefore increase climate impacts asymmetrically^7–9^. This phenomenon can in turn exacerbate structural inequalities^10^, potentially undermining the basic goal of urban integration^4,11,12^. To address these challenges, it is vital to improve our understanding of locally specific climate vulnerabilities and identify whether climate adaptations are responding to locally heterogeneous needs within urban agglomeration processes. Such work would also address the lack of research into the impacts of climate risk and climate adaptation policy at the local scale. Due to its massive scope and complexity, climate change tends to be investigated at the national or international scale^13^.

We use the Guangdong-Hong Kong-Macau Greater Bay Area (GBA), a site of major urban agglomeration led by the Chinese central government since at least 2008^14,15^, to examine the complexity and challenges of integrating existing local and municipal climate change policies. The GBA, which consists of two highly developed special administrative regions (Hong Kong and Macao) and nine economically dynamic cities within Guangdong province, is an ideal site for examining the effects of urban agglomeration because it demonstrates both the locally-generated and top-down dimensions of this process (Supplementary Figure S1). For instance, on the one hand, GBA cities experienced greater urban expansion than other primary cities in China, such as Beijing and Shanghai^16^. During the past decade, built-up land in the GBA increased by 19% while farmland decreased by 48%. On the other hand, the GBA’s agglomeration and integration is being centrally planned according to explicit policy initiatives that can be assessed with respect to their own stated goals, unlike in other regions where policy primarily reacts to ongoing agglomerative tendencies. As China seeks to become a global leader in both climate adaptation and urbanization policies^17,18^, understanding how they are playing out within the country's borders may inform assessments of the policies' potential for international export and implementation. The main framework guiding the GBA’s integration, the Outline Development Plan for the Guangdong-Hong Kong-Macao Greater Bay Area^19^, aims to realize “green development and ecological conservation” at the regional scale^20^.The document also expresses the need to “actively adapt to climate change,” (p. 35)—a goal that is in line with the broader conception of climate adaptation as involving sustainable and stable development^21^.

To identify whether the GBA’s environmental policies are accommodating local environmental and economic heterogeneity, we perform a spatial comparison between climate impacts, vulnerability, and adaptation components at the spatial (pixel-level) and temporal (yearly) scales^22^. We define greenspace as a major component of climate adaptation, as greenspace is explicitly listed as relating to climate adaptation within the GBA’s policy framework^20^. Historically in the GBA, engineered infrastructure projects like sea walls and dikes have been used to control the impacts of flooding, storm surges, and sea level rise. Most of these structures were built during the initial period of urban growth in the twentieth century, prior to climate change mitigation efforts. By contrast, in recent years, urban greenspace has been increasingly used for local climate adaptation because it effectively provides mitigation and adaptation functions, as well as other environmental and aesthetic benefits^23–25^. In regional planning efforts, GBA cities now often promote low-carbon strategies involving greenspace to strengthen their adaptation capacity^26^.

Technological and methodological advances combining satellite imagery with machine learning are helping to overcome a lack of sufficient information regarding climate change at the local scale^27^. Leveraging these innovations, we create novel climate impact and vulnerability indices to analyze the spatial correlation between greenspace increases for climate adaptation and climate impacts/vulnerability. We use these correlations as a proxy to measure climate policy effectiveness within an area that is both experiencing and being targeted for urban agglomeration. Our indicators to measure climate vulnerability are selected from relevant studies of cities in subtropical climate regions^28–30^ and further screened through an expert review process (Supplementary Table S1). The GBA exemplifies many of the impacts experienced by such cities, as it is highly exposed to typhoons, flooding, and intensive precipitation and high temperatures during the monsoon season. More specific to the local topography, climate impacts in the GBA are sensitive to elevation, distance to coastline, slope, the distribution of population, intensity of urbanization, and the share of the population that is less than 15 years old or older than 65 years old. We include these indicators to calculate a climate impact index. Finally, local economic status, existing green and gray infrastructure, accessibility to major road and public transportation, educational attainment, and health service capacity can also influence local adaptive capacity to climate change. Thus, we include these adaptive capacity indicators and combine them with the aforementioned climate impact indicators to construct a climate vulnerability index (Supplementary Tables S1–S2). Using the Analytic Hierarchy Process (AHP), we index each indicator at the smallest available unit (30-m resolution) from multiple satellite imagery datasets (Methods). To address the subjective nature of assigning weights of relative importance among the assessed indicators by expert judgement according to the AHP method, we also perform the fuzzy AHP technique based on the triangular membership function (Supplementary Figure S6). This allows us to measure the spatial heterogeneity of climate impacts and vulnerability at the local scale, thereby providing an evidentiary basis for measuring the spatial efficacy of local-scale climate policy within wider processes of urban agglomeration.

To validate the hypothesis that the greenspace is used by government agencies in the GBA to address climate impacts, we employ a logistic regression model with several diagnostic statistics (Methods). If there is no association between greenspace increase and climate impact indicators, this is taken to imply that the spatial effects of greenspace on climate vulnerability are not meaningful.

## Results

We find that the climate impact index and most of the adaptive capacity indicators are sensitive to greenspace increases (Table 1). After 2010, climate impacts are associated with an increase in greenspace. Gross Domestic Product (GDP) per capita, proximity to gray infrastructure, and accessibility to major roads are among the adaptive capacity indicators positively impacting land conversion to greenspace throughout our study period.

**Table 1:** Results of logistic regression for all hazards (impact threshold).

Aspect	Period	(1)		(2)		(3)	
		2005–2010		2010–2015		2015–2020	
		β	OR	β	OR	β	OR
Exposure	Typhoon frequency	− 0.039	0.962	0.074**	1.076**	0.027**	1.028**
	Flooding	− 0.025*	0.975*	0.022***	1.022***	0.050***	1.051***
	Precipitation	− 0.078	0.925	0.036***	1.037***	0.068**	1.070**
	Temperature	− 0.019**	0.981**	0.052***	1.054***	0.044**	1.045**
Sensitivity	Elevation	0.050**	1.051**	0.903	2.466	− 0.160***	0.852***
	Coastal proximity	− 0.042***	0.958***	− 0.027**	0.973**	− 0.023***	0.977***
	Slope	0.086***	1.090***	0.027***	1.027***	0.045***	1.046***
	Population density	− 0.057**	0.944**	0.067***	1.069***	0.031	1.032
	Urbanization density	− 0.063***	0.939***	0.025***	1.025***	0.032***	1.033***
	Vulnerable population	− 0.077***	0.926***	0.009	1.009	− 0.016	0.984
	Gender	− 0.022	0.978	− 0.008	0.992	− 0.008	0.992
Adaptive capacity	GDP per capita	0.122**	1.130**	0.044**	1.045**	0.161**	1.175**
	Gray infrastructure	0.042***	1.043***	− 0.035***	0.965***	− 0.017**	0.983**
	Green infrastructure	0.023**	1.023**	− 0.109	0.897	− 0.074	0.928
	Road accessibility	0.082**	1.086**	− 0.063**	0.939**	− 0.013***	0.987***
	Public transportation	0.047	1.048	− 0.026	0.975	− 0.034**	0.966**
	Educational attainment	0.016***	1.016***	0.059*	1.061*	0.029**	1.029**
	Hospitals	− 0.078	0.925	0.287	1.332	0.036**	1.037**
	Constant	2.900**	18.182**	− 6.056***	0.002***	− 2.501**	0.082**
	Observation	− 0.039***		2,667,128		1,744,423	
	ROC	− 0.025***		0.8522		0.8697	
	Pseudo *R*^2^	− 0.078		0.2564		0.3271	

Dependent variables are change to greenspace (forest, grassland or wetland) from all other land cover types (dummy = 1 if a cell changes to greenspace; 0 otherwise); β = coefficient of independent variables; OR (Odds Ratio) indicates the change in the odds ratio associated with a unit change in the predictor variable; ROC (Relative Operating Curve) is a measure of independent variables’ interpretability on the dependent variable. Standard errors are clustered at the township level; *p < 0.10, **p < 0.05, ***p < 0.01.

The results show, first, that greenspace has increasingly been used to mitigate climate impacts (exposure and sensitivity) over the past ten years (Table 1—columns 2 and 3). Second, regional-level green adaptation programs such as the Pearl River Delta Green Way Network program, established in 2010 in Guangdong province, and the Grassland Ecological Protection program, established in 2011 at the national level (including all of the GBA) (Supplementary Table S3), may have contributed to greenspace increases. To validate the greenspace variation trend between pre-initiative and post-initiative periods of these two greenspace programs, we perform logistic regressions individually by each hazard type (Supplementary Table S12). We find that the trend is broadly similar between the specification of the composite hazard and the separately analyzed set.

To examine the spatial distribution of greenspace across the dimensions of climate impacts and vulnerability, we cluster the normalized value of each index at the township level, the smallest unit of policy implementation within the GBA (comprising 1,381 townships), and reclassify them on a scale of 1 to 5, with 1 being the least greenspace and 5 being the most. We thus construct two unique indices: an impact-greenspace index and a vulnerability-greenspace index. These indices are calculated based on the spatial discrepancy between the indexed values of climate impacts/vulnerability and greenspace increases. For example, if the vulnerability index value is the same as greenspace index, the vulnerability-greenspace index value equals zero (no discrepancy). Similarly, if the vulnerability index value equals 5 (most vulnerable to climate change) while the greenspace increase rate is very low (index value equals 1), the vulnerability-greenspace index value equals 4 (highest discrepancy), meaning that greenspace has not increased in the areas where climate vulnerability is very high.

**Figure 1:**
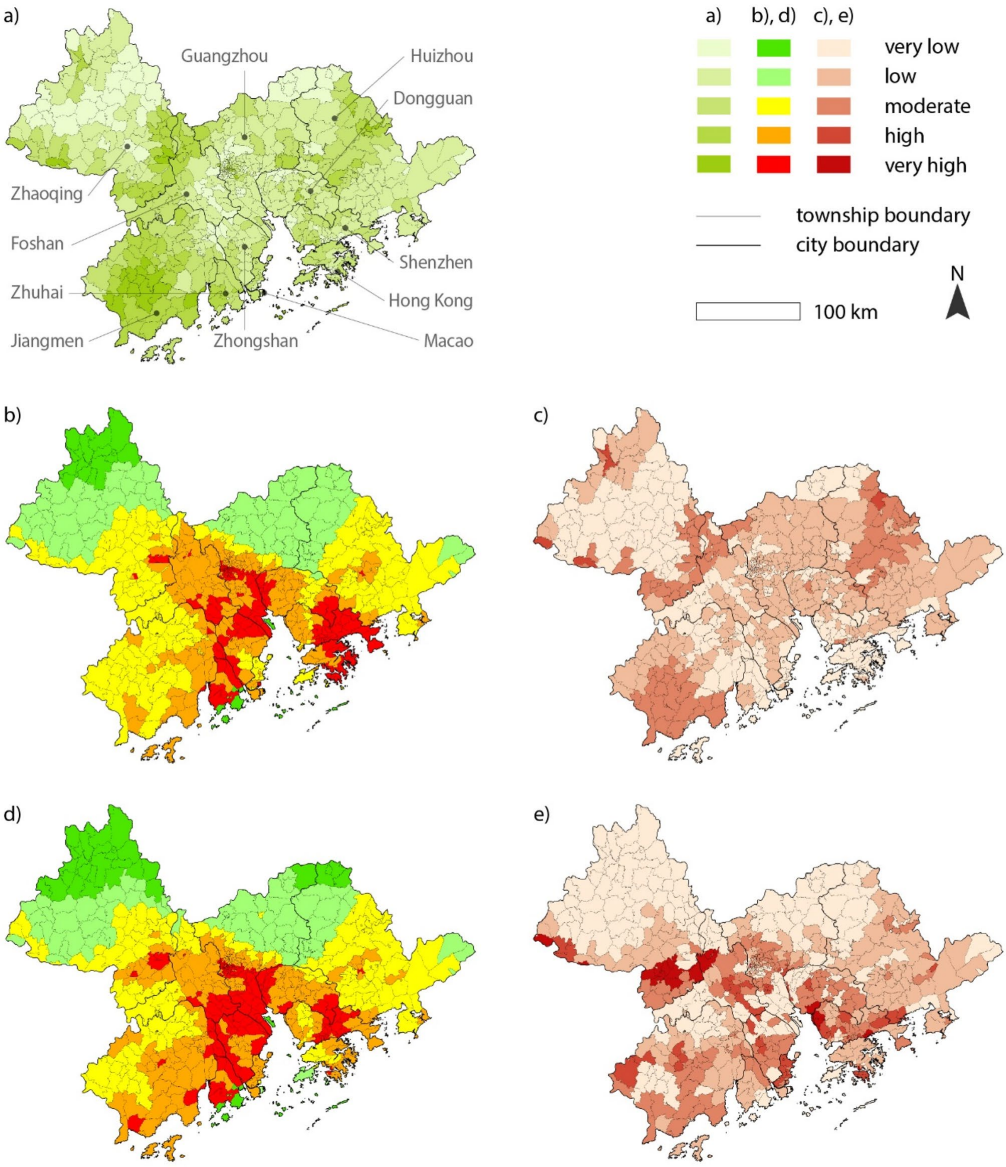
Impact-greenspace and vulnerability-greenspace index maps: (**a**) greenspace increase; (**b**) climate impacts; (**c**) impact-greenspace index; (**d**) climate vulnerability; and (**e**) vulnerability-greenspace index. Illustrated by authors using ArcMap software (Esri Inc. (2019). ArcMap 10.8, https://www.esri.com/en-us/arcgis).

**Figure 2:**
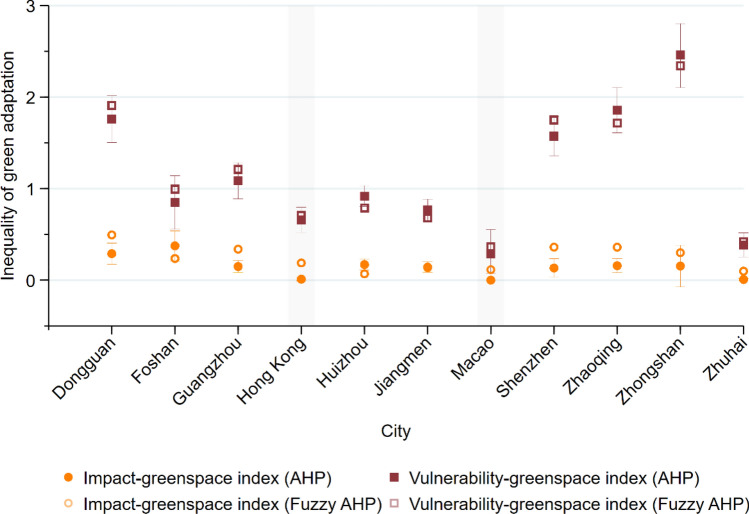
City-level spatial inequalities of green adaptation on climate impacts and vulnerability. Illustrated by authors using Stata statistical software (StataCorp LLC. Stata/MP 16, https://www.stata.com).

Here, we reveal that greenspace increases proportionately to the intensity of climate impacts (exposure + sensitivity) (Figs. 1c and 2). By contrast, many cities exhibit spatial discrepancies between greenspace variation and climate vulnerability (exposure + sensitivity − adaptive capacity) (Figs. 1e and 2). This result suggests that only 2.1% of the GBA townships exhibit a high discrepancy between greenspace increases and climate impacts (above the moderate level), while 30.6% exhibit a high discrepancy between the provision of green adaptation and climate vulnerability (Fig. 1). Although there is a slight difference in the degree of inequalities at the local scale due to marginal differences in the applied weights (Supplementary Table S11), these trends are broadly the same in the analyses based on AHP and fuzzy AHP with marginal differences (Fig. 2 and Supplementary Figure S5). The findings from these geospatial analyses further confirm the spatially uneven provision of green adaptation as a response to climate change across the GBA.

We find a basic mismatch between the green adaptation efforts that are being undertaken and actual needs based on climate vulnerability. This spatial inequality of green adaptation differs significantly across the GBA’s cities (Fig. 2). Hong Kong and Macao are relatively equal in their use of greenspace for mitigating climate vulnerability, while the other nine cities located in mainland China vary considerably. Foshan, Jiangmen, Zhuhai use greenspace relatively effectively for climate vulnerability. By contrast, a high level of spatial inequality of green adaptation on climate vulnerability is found in Zhongshan, Zhaoqing, Dongguan, and Shenzhen. Notably, the two cities with relatively high spatial inequality (Zhongshan and Shenzhen) are the ones participating in the central government’s eco-city program^31^.

The results of the logistic regression for green adaptation inequalities reveal that economic status is sensitive to greenspace increases. A higher GDP per capita is associated with a 4.1% and a 4.7% decrease in the odds of green adaptation inequality on climate impacts and vulnerability, respectively (Table 2). Higher elevation and higher health service capacity are associated with an increased probability of greenspace. However, there is no evidence that other indicators increase the discrepancy between greenspace variation and climate impacts at the 5% level of significance (Table 2—Column 1). Greater typhoon frequency, higher flood risk, higher temperatures, a steeper average slope, denser population, and greater distance from major roads increase green adaptation inequality on climate vulnerability, too (Table 2—column 2). The results suggest that areas with lower GDP and higher climate impacts are more likely to exhibit inequality in green adaptation.

**Table 2:** Results of logistic regression for green adaptation inequalities (2010–2020).

Aspect	Dependent variable	(1)		(2)	
		Green adaptation inequality on climate impacts		Green adaptation inequality on climate vulnerability	
	Indicator	β	OR	β	OR
Exposure	Typhoon frequency	− 0.021**	0.979**	0.081***	1.084***
	Flooding	− 0.040**	0.961**	0.023**	1.023**
	Precipitation	− 0.037**	0.963**	0.029	1.030
	Temperature	− 0.030	0.971	0.015***	1.015***
Sensitivity	Elevation	0.038***	1.039***	− 0.047***	0.954***
	Coastal proximity	0.020	1.021	− 0.038**	0.963**
	Slope	− 0.012***	0.988***	0.011***	1.011***
	Population density	− 0.028	0.973	0.021**	1.022**
	Urbanization density	− 0.015***	0.986***	0.027	1.027
	Vulnerable population	0.021*	1.021*	0.160	1.173
	Gender	− 0.024	0.976	0.092	1.096
Adaptive Capacity	GDP per capita	− 0.042***	0.959***	− 0.048***	0.953***
	Gray infrastructure	− 0.039	0.962	0.085	1.089
	Green infrastructure	− 0.036	0.965	− 0.008**	0.992**
	Road accessibility	0.024	1.024	0.030**	1.030**
	Public transportation	− 0.012	0.988	0.013	1.013
	Educational attainment	− 0.018	0.982	− 0.071**	0.931**
	Hospitals	0.020**	1.020**	0.116	1.123
	Constant	2.670	14.443	− 8.456***	0.002***
	Observation	2,666,968		2,666,968	
	ROC	0.743		0.848	
	Pseudo *R*^2^	0.137		0.328	

β = coefficient of independent variables; OR (Odds Ratio) indicates the change in the odds ratio associated with a unit change in the predictor variable; All independent variables are standardized values calculated by Eq. (2) (Methods); ROC (Relative Operating Curve) is a measure of independent variables’ interpretability on the dependent variable; Standard errors are clustered at the township level; *p < 0.10, **p < 0.05, ***p < 0.01.

Greenspace has increased in areas where overall climate exposure and sensitivity are high. Still, one third of townships within the GBA exhibit a spatially unequal use of greenspace as a means of reducing climate vulnerability within their boundaries (Fig. 1 and Supplementary Figure S5). Greater amounts of greenspace have been developed in wealthier communities regardless of existing vulnerability levels. By contrast, such adaptations have not been enacted to the same degree in poorer communities where climate vulnerability is high.

## Discussion

Climate adaptation can be viewed as a private good or a toll/club good, benefiting only individuals or communities investing in climate change adaptation^32–34^. On the one hand, creating greenspace can reduce the negative impacts caused by climate change by reducing temperature and flash floods, benefiting all nearby residents. On the other hand, privately initiated greenspace provision typically fails to generate such public utility^35^. In this aspect, local authorities play an influential role in determining how tax revenue is used to finance climate adaptation projects^36^. Problematically, decision-making and policy implementation processes often prioritize adaptation projects in areas that pay more taxes^4^. Subsequent inequalities in climate adaptation may thus arise, further marginalizing poorer communities where adaptation capacity is low^37^.

Spatial discrepancies between greenspace increases and climate vulnerability are observed more within the GBA cities located in mainland China. While Hong Kong and Macao continue to have their own local climate adaptation policies, the centrally-planned climate and environmental policies being implemented across heterogeneous climate vulnerability zones in Guangdong are limiting townships’ abilities to adapt to climate change. Despite significant differences at the township level in climate vulnerability across the GBA, the central government seeks to link its environmental policies and urban development goals, as emphasized in the GBA’s development framework^38^. The strategy document prioritizes ecology and green development, following the national roadmap for sustainable development^39^. Although various cities within the GBA are constructing additional greenspace as a means of addressing climate change, no cohesive adaptation action plan has yet emerged to integrate local heterogeneity in climate vulnerability within centralized urban agglomeration processes. This policy gap may lead climate adaptation to be inefficient and unevenly distributed by funneling adaptive resources into places where they are relatively less lacking.

Our results provide empirical evidence for the ways in which urban agglomeration policy can generate spatial inequalities in climate adaptation. Although climate adaptation is not likely to occur without strong government policies^40^, those that are insensitive to local needs can generate a vicious cycle that threatens to reduce the effectiveness of adaptation initiatives and increase social inequality^4^, undermining local sustainability. Our paper contributes to both climate policy and the urban planning literature by providing an identification methodology that can uncover the dilemmas provoked by centralized urban planning.

## Conclusion

By analyzing recent changes in the GBA, our study offers new insights and evidence to wider discussions about environmental justice and climate change adaptation. To investigate the impacts of urban agglomeration policy on climate adaptation, we exploit variation in greenspace across climate impacts and vulnerability dimensions in the GBA using various satellite imagery datasets and logistic regression.

Our approach to evaluating the spatiotemporal effects of urban planning, which scrutinizes the local effectiveness of climate adaptation, is novel in several respects. First, unlike past studies, this research focuses on the spatial inequality of green adaptation, which crucially influences urban sustainability at the township scale. Second, this study employs a cutting-edge method—land cover classification and a spatiotemporal identification of key climate vulnerability indicators using GEE and machine learning techniques—to preserve local-level sensitivity within a megalopolis. Third, we establish a unique set of identification indices (i.e., impact-greenspace and vulnerability-greenspace indices) to detect the localized spatial effects of regional urban policy.

We have examined spatial inequality in climate adaptation within a fast-agglomerating megalopolis in a tropical monsoon climate. As the spatial effects we identified likely vary across different climate conditions, future studies should consider these processes in the dry, temperate, and continental climates where many other cities are located. Moreover, particularly in countries with strong central or unitary governments pursuing urban development such as the United Kingdom and France, another compelling research direction would be for studies of climate justice to evaluate spatiotemporal correlations between climate adaptation and vulnerability with respect to the scale at which such policies are conceptualized. While municipal governments are at the vanguard of global climate change mitigation efforts like the C40 Cities Climate Leadership Group, their ability to act is often ultimately determined by national governments.

The empirical analysis offered herein suggests that urban agglomeration policy that fails to accommodate local heterogeneity can exacerbate spatial inequalities in climate adaptation. First, we find that regional-level green adaptation initiatives contribute to mitigating climate impacts. Second, urban agglomeration policy positively impacts climate adaptation against climate exposure and sensitivity, but negatively affects spatial equity in the provision of climate adaptation. Lastly, green adaptation tends to funnel into wealthier, less vulnerable areas while bypassing more vulnerable ones, increasing the potential impacts of climate change and undermining the benefits of urban agglomeration. The results suggest that in order to enhance urban sustainability, centrally-planned climate adaptation policy must accommodate local heterogeneity.

## Materials and methods

We combine Google Earth Engine (GEE)-based remote sensing techniques and climate vulnerability assessments with the Analytic Hierarchy Process (AHP) to examine the spatiotemporal correlation between greenspace and climate impacts/vulnerability. Using the logistic function, we identify the spatial inequality of green adaptation across the dimensions of climate impacts and vulnerability.

### Land cover change assessment

To identify greenspace changes in the GBA, we use cloud-free Landsat images from the United States Geological Survey (USGS) in GEE. More specifically, we use imagery from the 30-m Tier 1 Raw Scenes dataset from Landsat 5 Thematic Mapper (TM) and Landsat 8 Operational Land Imager/Thermal Infrared Sensor (OLI/TIRS) Collection, which are inter-calibrated across the different Landsat sensors. Given the GBA's humid subtropical monsoon climate, the growing season is all year-round. Thus, we select the median of the pixels from winter season images (November–February) from 2005, 2010, 2015, and 2020, using the Simple Composite algorithm based on the WGS-84 Coordinate System.

Based on the USGS National Land Cover Database (NLCD) and the Data Center for Resources and Environmental Sciences at the Chinese Academy of Sciences (RESDC)’s localized classifications, we identify seven land cover types: urban (built-up), forest (woodland), farmland (cropland and orchard), grassland, wetland, barren (unused land), and water bodies^41–43^. Although orchard land is often included for local-scale land cover studies, it is not necessary for our purposes because orchards are considered as farmland in the sampled area. Furthermore, separating orchards from cropland would reduce the reliability and accuracy of the classification. We use the hybrid method of unsupervised (using Weka K-Means clustering algorithm) and supervised machine learning to increase the accuracy and consistency of the time-varying classification^44,45^. The Support Vector Machine (SVM) method with the manually defined land classification samples is used to train a total of 30 unsupervised land cover groups in each map in each classifying year. The training sample polygons of each classification in each year for SVM are digitized based on high-resolution historical satellite images from Google Earth Pro. To test the accuracy of our land cover classification, we employ a confusion matrix using stratified random sampling. The overall accuracy and Kappa coefficient of the maps in all years are higher than 0.93 and 0.89, respectively (Supplementary Table S4), indicating the classification is reliable, with substantial agreement among the individual collecting data^46^. The proportion of each land cover category resembles the land cover statistics provided by RESDC. The land classification map in each year is shown in Supplementary Figure S2, and the JavaScript code for the land cover classification is available in Supplementary Codes S1-S2.

Using the classified maps, we spatially quantify land cover changes from 2005–2010, 2010–2015, and 2015–2020. In order to specify the spatial attributes of these changes, we generate a center point for each raster grid (30-m resolution) covering the entire GBA and overlay it onto the classified land change maps. The centroid points contain geospatial coordinate information. Subsequently, we compare changing patterns of land cover, which are associated with climate adaptation factors such as distance to coastlines, slopes, and elevation. The land cover change matrix equation is as follows:

1 $$C_{ijk} = \frac{{\delta_{ijk} }}{{\mathop \sum \nolimits_{j = 1}^{n} \delta_{ik} }} \times 100\%$$

where $\delta_{ijk}$ represents the transition area from land cover type *i* at the beginning to land cover type *j* at the end in grid *k*, with *n* for the number of land cover types. $C_{ijk}$ indicates the percentage of transformed land cover *j* at the end from the initial land cover *i* in grid *k*. The overall spatiotemporal land cover trends from 2005–2020 are illustrated in Supplementary Figures S3 and S4.

### Climate change indicators

To define climate change impacts, we adopt the Intergovernmental Panel on Climate Change (IPCC)’s conceptual framework of vulnerability, defined as a function of exposure, sensitivity, and adaptive capacity^47–49^. Based on previous literature conducting vulnerability assessments in subtropical climate regions in Asia^28–30,50^, America^51–53^, and Europe^48,54^ and our own screening process with expert groups (Supplementary Table S1), we select measurable and locally critical indicators for each of these three functions.

Our study area is characterized by a subtropical monsoon climate, which means tropical cyclones are a regular occurrence. The China Meteorological Administration predicts that sea level in the Pearl River Delta will rise by 78–150 mm between 2008 and 2038^55^. The Hong Kong Climate Change Report (2015) indicates that annual precipitation may rise by 180 mm by the end of the twenty-first century, while the chance of a daily maximum temperature higher than 35°C increases to 22% in the early twenty-first century compared with the 3% in early twentieth century^56^. Coupled with rapid urbanization, the GBA may thus become more vulnerable to flood risk^57^. Thus, we employ four indicators to measure exposure: typhoon frequency, flood risk, precipitation, and temperature.

Sensitivity, or the degree to which a system is affected by climate change, is largely a result of physical characteristics including elevation, coastal proximity, slope, population density, and urbanization density^28,30,54^. Sensitivity is also affected by social characteristics such as a population’s vulnerability^28,50–54^ and gender^51,54^ ratio. Marginalized populations, including children, elderly people, and women, are often at greater risk in disasters^58^. Thus, we include a total of seven major indicators for sensitivity.

Adaptive capacity, or the ability of a system to deal with climatic extremes, is broadly influenced by economic, social, knowledge-based, infrastructural, and institutional capacities^48,53,59^. Drawing on literature focused on the GBA, we further refined our adaptive indicators to suit the local context (Yang et al. 2015; Zhang and Chen 2019). We therefore include five indicators representing economic and infrastructural capacities: GDP per capita, gray infrastructure, green infrastructure, road accessibility, and public transportation. As a considerable number of studies^28, 51–54^ in other regions of the world also use level of education (bachelor’s degree or higher) and the number of medical institutions to measure adaptive capacity, we also include these two indicators. We thus use a total of seven indicators for adaptive capacity. The selected climate vulnerability indicators with descriptive statistics are shown in Supplementary Table S2.

To define the impact areas of each typhoon, we use the U.S. National Oceanic and Atmospheric Administration’s (NOAA) historical hurricane track data (the shortest radius of 30 knot winds or greater)^60^. Given that the World Meteorological Organization (WMO)’s tropical cyclone intensity scale defines the maximum sustained surface winds speed of a tropical storm as exceeding 34 knots, the average radius of 30-knot winds from a storm tract is used as the perimeter for estimating the most intensely affected storm impact areas. In GEE, we analyzed various satellite imagery datasets to identify flood risk areas, elevation, slope, and urbanization density (Supplementary Codes S3-S6). The flood risk areas that are inundated in the wet season are identified by applying the Normalized Difference Water Index (NDWI) function on the satellite imagery in each period^61^. Elevation and slope values of each pixel are extracted from Shuttle Radar Topography Mission (SRTM) digital elevation data. A night-time light index calculated from the Visible Infrared Imaging Radiometer Suite (VIIRS) Day Night Band (Version 1) Composites is used as a proxy for urbanization density^62^. The percent of the population deemed “vulnerable” (aged 0–14 or 65 and over)^54^, gender ratio, and level of educational attainment within each township are collected from official statistics from each government^63–65^. Geospatial coordinates and categories of hospitals to measure the health care capacity are extracted from Gaode Map.

We calibrated high-spatial-resolution monthly mean temperature and precipitation data^66^ with historical local weather records. Other indicators, administrative geospatial data, and socio-economic statistics for indicators of climate sensitivity and adaptive capacity were collected from OpenStreetMap and CEIC, a private company which provides macroeconomic data.

### Climate impacts and vulnerability assessment

In order to assess the spatial correlation between greenspace changes and the magnitude of climate impacts/vulnerability, we establish climate impact and vulnerability index maps. Using the defined indicators of climate change, we perform the following three steps to calculate the comprehensive weighted value of each pixel.

First, each climate vulnerability factor has different dimensions and orders of magnitude. In order to compare all values uniformly, it is necessary to normalize them so that each value is converted to a range between 0 and 1 by applying the linear Max–Min method (Eq. 2)^12,30^.

2 $$\delta_{ij} = \frac{{\chi_{ij} - {\text{min}}_{{{\text{ij}}}} }}{{{\text{max}}_{{{\text{ij}}}} - {\text{min}}_{{{\text{ij}}}} }}$$

where, $\delta_{ij}$ is a normalized value for the factor $i$ in pixel $j$; $\chi_{ij}$ is an original value of the factor, ${\text{min}}_{{{\text{ij}}}}$ and ${\text{max}}_{{{\text{ij}}}}$ are the minimum and maximum values, respectively.

Second, we use the AHP method, a multiple-criteria decision-making approach introduced by Saaty (1977)^67^, to weigh the normalized factors. This method, which is structured using sets of pair-wise comparisons, is widely used in natural disaster risk studies^30,68^. We used Saaty’s original comparative scale between 1 and 9, in which “1” suggests that two factors hold equal importance, while “9” is assigned when one factor is significantly more important than the other. In order to obtain the AHP weight values, we conducted a survey distributed from February 4–5, 2021 to experts through email/telephone interviews with 14 experts from four entities spanning the public sector, academia, and non-governmental research institutes: the Guangzhou Urban Planning Institute, Korean Ministry of Environment, Massachusetts Institute of Technology China City Lab, and Lincoln Institute of Land Policy for China Program. As the first author is familiar with the aforementioned institutes and organizations from previous collaborations and projects, we invited them to select experts with sufficient knowledge and experience in the fields of flood control, climate disaster mitigation, and/or climate change adaptation to participate in the our survey. While the respondents’ work experience ranged from six to 23 years, we chose not to weigh the initial judgements by this. The first-round expert judgements are based on separate individual hazards (typhoon, floods, and high temperatures) (Supplementary Tables S5–S7). The second judgement process took place from February 6–8, 2021 and involved nine experts from the same institutes as in the first round, as five of the original participants were unresponsive within the allotted timeframe. The second round was based on one generic set of indicators chosen from the results of the first round. The weights of indicators for all hazard types together are shown in Supplementary Table S8, while full survey results are shown in Supplementary Tables S13–S16. The consistency ratios in each AHP matrix are all less than 0.1, meaning that the criterion matrices are satisfactorily consistent.

Finally, we calculate a comprehensive weighted value (Supplementary Table S10) using the following equation:

3 $$\zeta_{j} = W_{E} \left( {\mathop \sum \limits_{f = 1}^{n} \omega_{f} E_{fj} } \right) \times \delta_{fj} + W_{S} \left( {\mathop \sum \limits_{g = 1}^{n} \omega_{g} S_{gj} } \right) \times \delta_{gj} - W_{C} \left( {\mathop \sum \limits_{h = 1}^{n} \omega_{h} C_{hj} } \right) \times \delta_{hj}$$

where $\zeta_{j}$ is a comprehensive weighted value in pixel $j$*;* $W_{E}$, $W_{S}$, and $W_{C}$ are the weights of exposure, sensitivity, and adaptive capacity, respectively; $\omega_{f}$, $\omega_{g}$, and $\omega_{h}$ are the weights of different indicators in the three vulnerability criteria; $E_{f}$, $S_{g}$, and $C_{h}$ is the *f*^th^, *g*^th^, and *h*^th^ indicator within the exposure, sensitivity, and adaptive capacity criteria in pixel $j$, respectively; and $\delta_{fj}$, $\delta_{gj}$, and $\delta_{hj}$ are the normalized values for different indicators in pixel $j$.

### Robustness check with Fuzzy AHP method

Since AHP method has certain limitations due to its reliance on subjective judgements by experts who determine the relative importance of various indicators, we further employ the fuzzy AHP technique adopted from Chang (1996) as a robustness check. Instead of using a crisp value in the pair-wise comparison in AHP, fuzzy AHP uses a range of values based on a triangular membership function to help reduce the uncertainty of human judgement^69,70^. To obtain fuzzy AHP weights, we use the following equations:

4 $$\widetilde{{S_{i} }} = \mathop \sum \limits_{j = 1}^{n} \tilde{a}_{ij} \left( {\mathop \sum \limits_{i = 1}^{n} \cdot \mathop \sum \limits_{j = 1}^{n} \tilde{a}_{ij} } \right)^{ - 1}$$

where, $$\widetilde{{S_{i} }}$$ is the fuzzy synthetic extent value; $$\tilde{a}_{ij}$$ = ($$l_{ij} , m_{ij} , u_{ij} )$$;  ($$(a_{ij})^{-1} = (\frac{1}{{u_{ij} }},\frac{1}{{m_{ij} }},\frac{1}{{l_{ij} }})$$; $$i$$ and $$j$$ = 1, 2, … , n; and $$i \ne j$$.

The probability degree of $\widetilde{{S_{i} }} \ge S_{j}$ is defined as $V\left( {\widetilde{{S_{i} }} \ge \widetilde{{S_{j} }}} \right) = \sup_{{{\text{y}} \ge {\text{x}}}} \left[ {{\text{min}}\left( {\widetilde{{S_{j} }}\left( x \right), \widetilde{{S_{i} }}\left( y \right)} \right)} \right]$, in which $\widetilde{{S_{i} }} = \left( {l_{i} , m_{i} , u_{i} } \right)$ and $\widetilde{{S_{j} }} = \left( {l_{j} , m_{j} , u_{j} } \right)$, and can be expressed as follows:

5 $$V\left( {\widetilde{{S_{i} }} \ge \widetilde{{S_{j} }}} \right) = \left\{ {\begin{array}{*{20}c} {1,} & {{\text{if }}m_{i} \ge m_{j} } \\ {0,} & {{\text{if }}l_{j} \ge u_{i} } \\ {\frac{{u_{i} - l_{j} }}{{\left( {u_{i} - m_{i} } \right) + \left( {m_{j} - l_{j} } \right)}},} & {{\text{otherwise}}} \\ \end{array} } \right.$$

where $\widetilde{{S_{i} }} \ge \widetilde{{S_{j} }}$ is the ordinate of highest intersection point between $m_{i}$ and $m_{j}$ (Supplementary Figure S6). Applying the criteria in the Eq. (5), the fuzzy AHP weight values are calculated using the following equation:

6 $$W_{i} = \frac{{\min V\left( {\widetilde{{S_{i} }} \ge \widetilde{{S_{j} }}} \right)}}{{\mathop \sum \nolimits_{k = 1}^{n} \min V\left( {\widetilde{{S_{k} }} \ge \widetilde{{S_{j} }}} \right))}}, i\ \text{and}\ j = 1, 2, \ldots , n; i \ne j; j \ne k$$

To express a pair-wise comparison among the assessed indicators, we use the following linguistic scale with triangular fuzzy numbers in parentheses: 1. Equal importance (1, 1, 1); 3. Moderate importance (2, 3, 4); 5. Strong importance (4, 5, 6); 7. Very strong importance (6, 7, 8); 9. Extreme importance (9, 9, 9). 2, 4, 6, 8 (x-1, x, x + 1) represent intermediate values. The full fuzzy AHP matrix is shown in Supplementary Table S9.

### Quantification of transition probability to greenspace

Our measure of land cover change has a binary value: it is either changed or unchanged and can thus be expressed by 1 or 0. We use a logistic function to estimate the effects of climate vulnerability indicators on land cover changes to greenspace (Eq. 7).

7 $$P\left( {y_{i} = 1{|}\chi_{i} } \right) = P\left[ {\varepsilon_{i} \le \left( {\alpha + \beta \chi_{i} } \right)} \right] = \frac{1}{{1 + e^{{ - \varepsilon_{i} }} }}$$

where $y_{i}$ is a binary dependent variable equal to 1 if land cover *i* is changed to greenspace and equal to 0 otherwise; the vector of covariates $\chi_{i}$ refers to indicators of climate exposure, sensitivity, and adaptive capacity; $\alpha$ is the intercept; $\beta$ is the regression coefficient; and $\varepsilon_{i}$ is the error term.

In all models, Hosmer–Lemeshow P-values have greater than 10% statistical significance, and overall accuracy rates in the classification table are higher than 80%. The diagnostic statistics (large p-values of goodness-of-fit test, a high classification accuracy, ROC, and pseudo R^2^) for all of our models across various time periods confirm that they meet the study demands^71–73^.

## Supplementary Information

Supplementary Information
