# Supplementary material for: Urban agglomeration worsens spatial disparities in climate adaptation
Source: Sci Rep. 2021 Apr 19;11:8446. doi: 10.1038/s41598-021-87739-1 (PMC8055703; doi:10.1038/s41598-021-87739-1)
Supplement: Supplementary file 1 — Supplementary Information [file 41598_2021_87739_MOESM1_ESM.docx]

**Urban agglomeration worsens spatial disparities in climate adaptation**

**Seung Kyum Kim^1^*, Mia M. Bennett^2^, Terry van Gevelt^3^, and Paul Joosse^4^**

1. Faculty of Social Science, University of Hong Kong

7.02C, Jockey Club Tower, Centennial Campus, Pokfulam Road, Hong Kong SAR skim1@hku.hk

1. Department of Geography and School of Modern Languages and Cultures (China Studies Programme), University of Hong Kong

10.37, The Jockey Club Tower, Centennial Campus, Pokfulam Road, Hong Kong SAR mbennett@hku.hk

1. Department of Politics and Public Administration, University of Hong Kong

948, The Jockey Club Tower, Centennial Campus, Pokfulam Road, Hong Kong SAR
tvgevelt@hku.hk

1. Department of Sociology, University of Hong Kong

916, The Jockey Club Tower, Centennial Campus, Pokfulam Road, Hong Kong SAR

pjoosse@hku.hk

**Supplementary Information**

**Supplementary Figures**

Supplementary Figure S1. Site map.


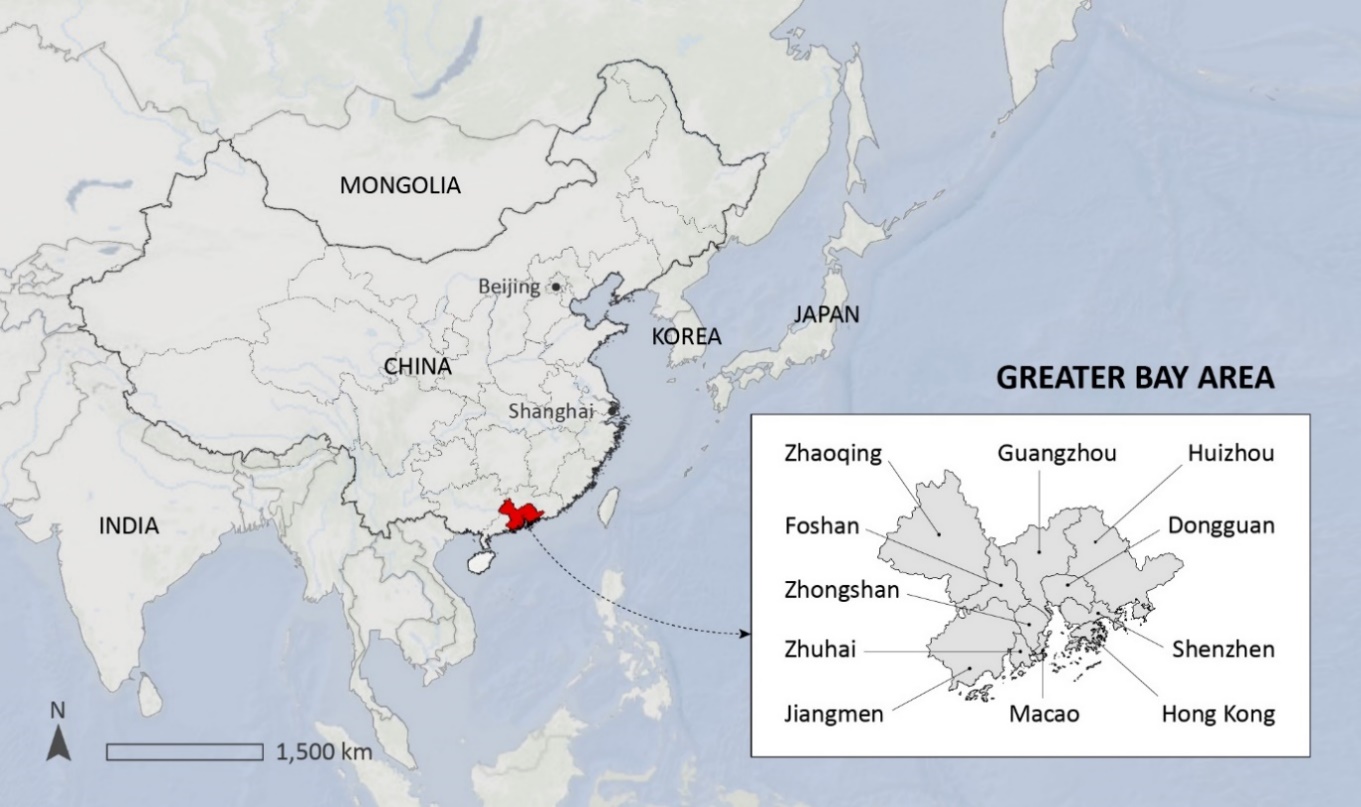


Notes: Illustrated by author using ArcMap software (Esri Inc. (2019). ArcMap 10.8, https://www.esri.com/en-us/arcgis).

Supplementary Figure S2. Land cover classification map in the GBA (2005 – 2020).


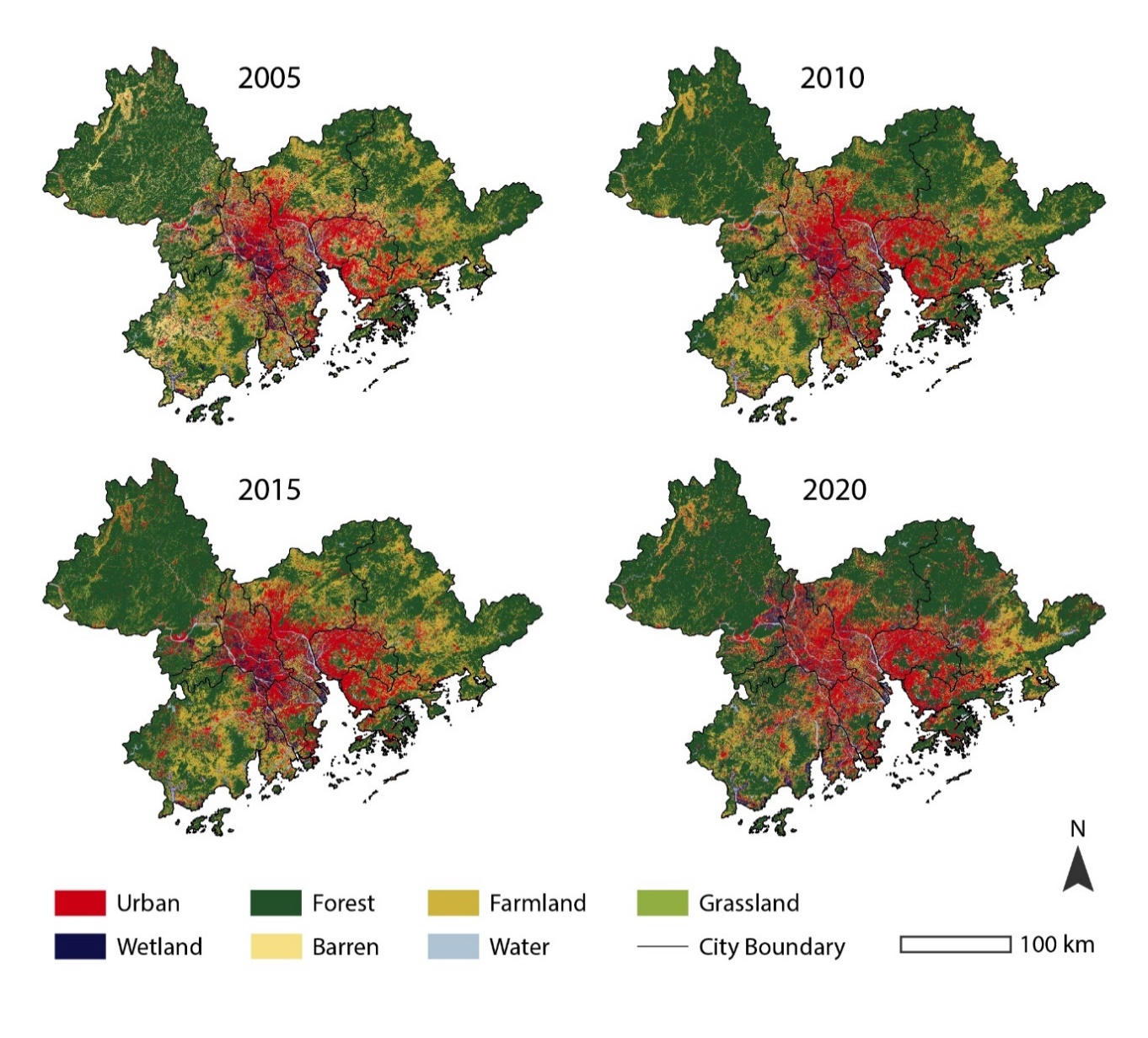


Notes: Illustrated by author using Google Earth Engine online computing platform (Gorelick, N., Hancher, M., Dixon, M., Ilyushchenko, S., Thau, D., & Moore, R. (2017). Google Earth Engine: Planetary-scale geospatial analysis for everyone. Remote Sensing of Environment, https://code.earthengine.google.com).

Supplementary Figure S3. Land cover proportion in different geographical areas in the GBA.

Notes: Proportion of each land cover are an average value between 2005 and 2020. Coastal area is defined as the areas where elevation is below 30 m within 20 km of coastline. Inland area refers to the areas further than 20 km from the coastline.

Supplementary Figure S4. Greenspace changes between coastal and inland areas over time.

Notes: Land cover change from non-conservation lands (urban, farmland, barren, and water) to conservation lands (forest, grassland, and wetland).

Supplementary Figure S5. Fuzzy AHP based impact-greenspace and vulnerability-greenspace index maps.


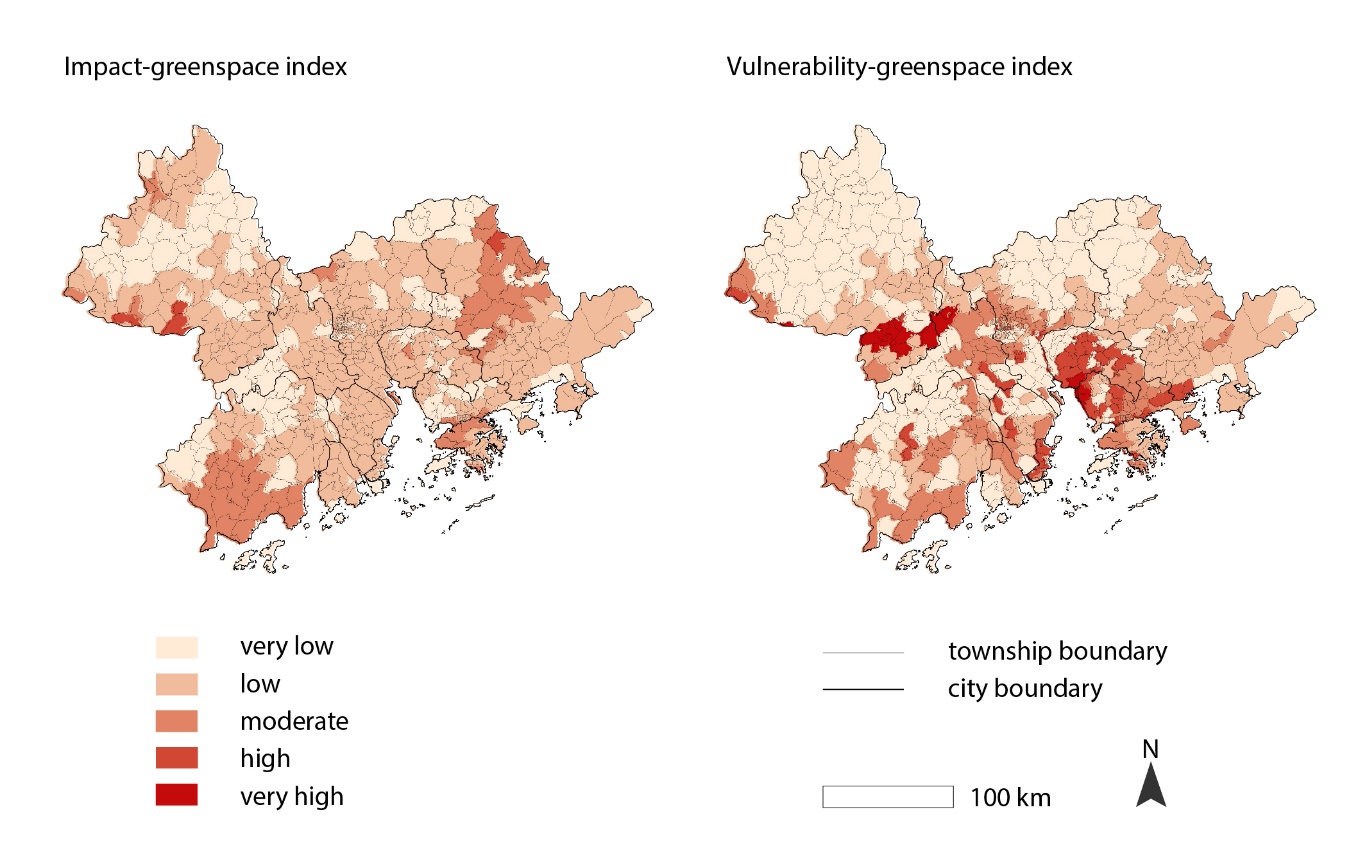


Notes: Illustrated by author using ArcMap software (Esri Inc. (2019). ArcMap 10.8, https://www.esri.com/en-us/arcgis).

Supplementary Figure S6. Fuzzy membership function.


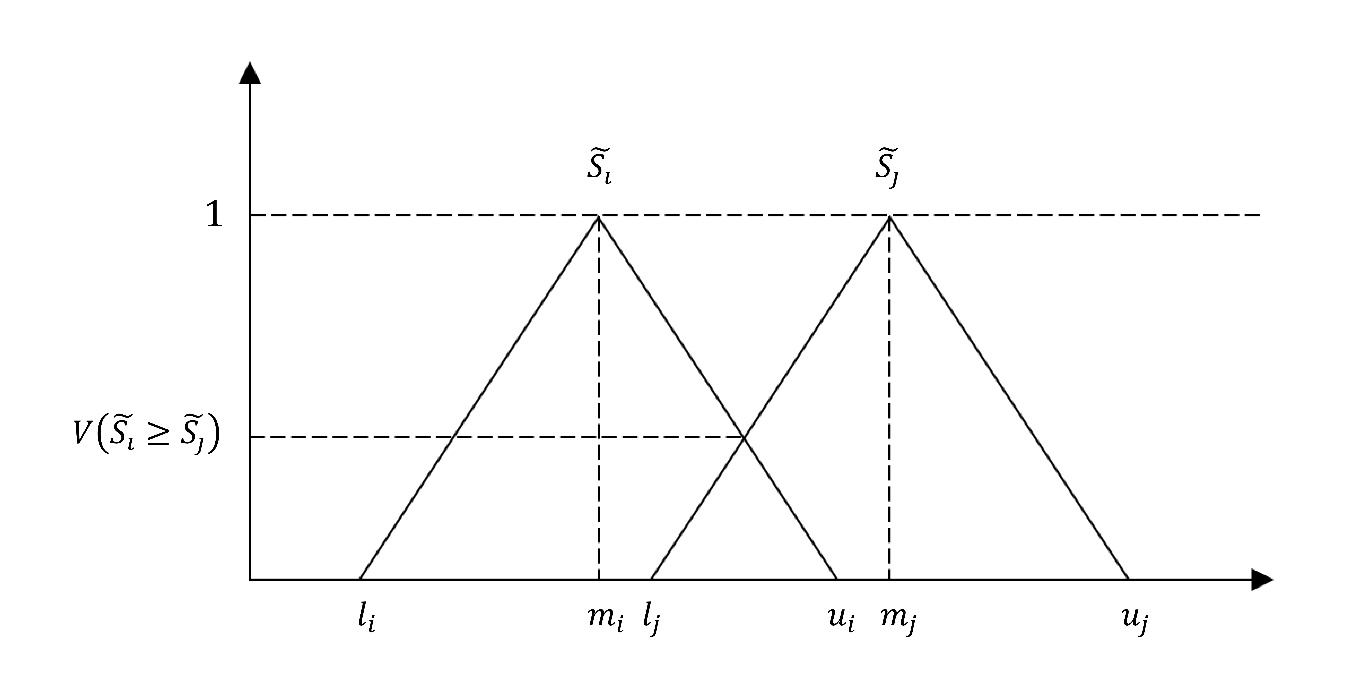


Notes: Illustrated by author using Adobe Illustrator (Adobe Inc. (2019). Adobe Illustrator CC 2018 (23.0.1), https://adobe.com/products/illustrator).

**Supplementary Tables**

Supplementary Table S1. Selection of indicators by expert groups.

| Aspects | Indicators | Sub-indicators | Typhoon | Flood | Temperature |
| --- | --- | --- | --- | --- | --- |
| Exposure | Typhoon frequency |  | 14 | 14 | 4 |
|  | Flood |  | 14 | 14 | 3 |
|  | Precipitation |  | 11 | 14 | 13 |
|  | Temperature |  | 2 | 3 | 14 |
| Sensitivity | Elevation |  | 14 | 14 | 3 |
|  | Coastal proximity |  | 14 | 6 | 6 |
|  | Slope |  | 14 | 14 | 4 |
|  | Population density |  | 14 | 14 | 13 |
|  | Urbanization density |  | 14 | 14 | 13 |
|  | Vulnerable population |  | 14 | 14 | 14 |
|  | Gender |  | 11 | 11 | 10 |
| Adaptive  Capacity | Economy | GDP per capita | 14 | 14 | 14 |
|  | Gray infrastructure | Canal | 14 | 14 | 2 |
|  |  | Ditch | 12 | 14 | 2 |
|  |  | Dam | 14 | 14 | 11 |
|  |  | Weir | 13 | 14 | 10 |
|  |  | Seawall | 14 | 5 | 0 |
|  | Green infrastructure | Lake and reservoir | 13 | 14 | 14 |
|  |  | Stormwater drainage system | 14 | 14 | 0 |
|  |  | Detention pond | 14 | 14 | 12 |
|  | Road accessibility |  | 14 | 12 | 14 |
|  | Public transportation |  | 14 | 13 | 14 |
|  | Education attainment |  | 12 | 12 | 12 |
|  | Knowledge | University | 8 | 8 | 6 |
|  | Health service capacity | Hospital | 14 | 14 | 14 |

Notes: European Spatial Planning Observation Network (ESPON)’s exposure and sensitivity indicators are used to define the links between the aspects and factors on vulnerability^48^. The numbers in the columns “Typhoon,” “Flood,” and “Temperature” are the sum of individual expert judgements on the selection of climate vulnerability indicators in this study. 14 experts were invited for the questionnaire from government and educational sectors, and from non-governmental research institutes (Korean Ministry of Environment, Guangzhou Urban Planning Institute, Massachusetts Institute of Technology’s China City Lab, and Lincoln Institution of Land Policy for China Program) between February 4 and 5, 2021. All participants have more than six years of experience in the fields of flood control, climate disaster mitigation, and/or climate change adaptation. The full questionnaire responses are shown in Supplementary Table S13-S16.

Supplementary Table S2. Selected climate vulnerability indicators and descriptive statistics.

| Aspects | Factors |  | Mean | S.D. | Min | Max |
| --- | --- | --- | --- | --- | --- | --- |
| Exposure | Typhoon frequency | Number of tropical cyclones (max wind > 40 knots) | 8.99 | 0.98 | 5.00 | 10.67 |
|  | Flood | 1 if a pixel is in a 100-year floodplain, 0 otherwise | 0.05 | 0.21 | 0 | 1 |
|  | Precipitation | Mean rainfall (wet season*, mm) | 346.35 | 46.79 | 177.75 | 473.75 |
|  | Temperature | Mean maximum temperature (wet season^a)^, Celsius) | 32.96 | 3.34 | 10.81 | 39.06 |
| Sensitivity | Elevation | Elevation above sea-level (m) | 130.13 | 172.21 | -55 | 1593 |
|  | Coastal proximity | Distance to coastline (km) | 77.35 | 62.32 | 0 | 236.86 |
|  | Slope | Slope degree | 7.54 | 7.53 | 0 | 53 |
|  | Population density | Population density per hectare | 9.45 | 18.63 | 0 | 216.57 |
|  | Urbanization density | Nighttime light index^b)^ | 4.60 | 8.81 | 0 | 63 |
|  | Vulnerable population | Percentage of population 0-14, 65 years old and above in township | 0.26 | 0.09 | 0.01 | 0.46 |
|  | Gender | Percentage of female population in township | 0.48 | 0.02 | 0.31 | 0.56 |
| Adaptive  Capacity | Economy | GDP per capita (1,000 US$) | 12.33 | 8.40 | 4.35 | 87.21 |
|  | Gray infrastructure | Distance to canal, ditch, dam, weir, and seawall (km) | 9.25 | 8.63 | 0 | 50.69 |
|  | Green infrastructure | Distance to lake, reservoir, detention pond, and stormwater drainages (km) | 1.44 | 1.73 | 0 | 25.08 |
|  | Road accessibility | Distance to major road (km) | 1.21 | 1.43 | 0 | 24.73 |
|  | Public transportation | Distance to subway station (km) | 20.99 | 18.21 | 0.01 | 102.22 |
|  | Education attainment | Percentage of population with bachelor’s degree or higher in township | 0.03 | 0.04 | 0.01 | 0.35 |
|  | Health service capacity | Number of hospitals in township | 1.98 | 2.68 | 0 | 17 |

Notes: Observations: 2,685,207. a) Wet season is defined as months between June and August. b) Average values of monthly VIIRS/DNB between 2012-2015 and 2015-2019 for 2005-2010 and 2015-2020 timeframes, respectively. For 2005-2010 timeframe, we use a penalty applied (using comparison between 2005, 2006, 2007, 2008, 2009, and 2010 with an average value of monthly VIIRS/DNB in 2012, because VIIRS/DNB was launched in 2012.

Supplementary Table S3. Major regional development policies in the GBA since 2000.

| Year | Name |
| --- | --- |
| 2001 | Urban agglomeration development planning in the Pearl River Delta |
| 2004 | PRD Urban Cluster Coordinated Development Plan (2004–2020) |
| 2008 | Outline of the Plan for the Reform and Development of the PRD (2008–2020) |
| 2009 | The key action planning for the construction of the livable Bay Area in the Pearl River Estuary |
| 2009 | Integrated planning for urban and rural development in the Pearl River Delta |
| 2009 | Special planning for building a high quality life circle |
| 2010 | Outline of overall planning for greenway network in Pearl River Delta |
| 2011 | National Grassland Ecological Protection Program |
| 2014 | The PRD Region Plan (2014–2030) |
| 2019 | Outline Development Plan for the Guangdong–Hong Kong–Macao Greater Bay Area |

Source: Liang, Y., Zhou, Z., & Li, X. (2019). Dynamic of Regional Planning and Sustainable Development in the Pearl River Delta, China. *Sustainability*, 11(21), 6074. Sun et al. (2018). China’s progress towards sustainable land development and ecological civilization. *Landscape Ecology,* 33, 1647-1653.

Supplementary Table S4. Accuracy assessment for classified images.

| Map (Year) | Overall Accuracy | Kappa Coefficient |
| --- | --- | --- |
| 2005 | 0.937 | 0.896 |
| 2010 | 0.953 | 0.919 |
| 2015 | 0.958 | 0.928 |
| 2020 | 0.966 | 0.945 |

Notes: Confusion Martix (Stehman 1997) is used to assess the accuracy of classifier using Google Earth Engine. land cover classification.

Supplementary Table S5. AHP matrix of the vulnerability indicators for typhoon.

| Aspect | A1 | A2 | A3 |  |  |  |  | Weight |
| --- | --- | --- | --- | --- | --- | --- | --- | --- |
| Exposure (A1) | 1 | 3 | 4 |  |  |  |  | 0.614 |
| Sensitivity (A2) | 1/3 | 1 | 1 |  |  |  |  | 0.203 |
| Adaptive Capacity (A3) | 1/4 | 1 | 1 |  |  |  |  | 0.183 |
|  |  |  |  |  |  |  |  |  |
| Exposure | E1 | E2 | E3 |  |  |  |  | Weight |
| Typhoon (E1) | 1 | 2 | 3 |  |  |  |  | 0.526 |
| Flood (E2) | 1/2 | 1 | 2 |  |  |  |  | 0.312 |
| Precipitation (E3) | 1/3 | 1/2 | 1 |  |  |  |  | 0.162 |
|  |  |  |  |  |  |  |  |  |
| Sensitivity | S1 | S2 | S3 | S4 | S5 | S6 | S7 | Weight |
| Elevation (S1) | 1 | 1 | 1 | 2 | 2 | 3 | 4 | 0.215 |
| Coastal proximity (S2) | 1 | 1 | 1 | 3 | 3 | 3 | 4 | 0.230 |
| Slope (S3) | 1 | 1 | 1 | 3 | 3 | 4 | 5 | 0.244 |
| Population density (S4) | 1/2 | 1/3 | 1/3 | 1 | 1 | 1 | 2 | 0.082 |
| Urbanization density (S5) | 1/2 | 1/3 | 1/3 | 1 | 1 | 1 | 2 | 0.079 |
| Vulnerable population (S6) | 1/3 | 1/3 | 1/4 | 2 | 2 | 1 | 2 | 0.101 |
| Gender (S7) | 1/4 | 1/4 | 1/5 | 1/2 | 1/2 | 1/2 | 1 | 0.049 |
|  |  |  |  |  |  |  |  |  |
| Adaptive Capacity | C1 | C2 | C3 | C4 | C5 | C6 | C7 | Weight |
| GDP per capita (C1) | 1 | 3 | 2 | 4 | 4 | 4 | 1 | 0.282 |
| Gray Infrastructure (C2) | 1/3 | 1 | 1 | 3 | 3 | 2 | 1/2 | 0.143 |
| Green Infrastructure (C3) | 1/2 | 1 | 1 | 3 | 3 | 2 | 1/2 | 0.145 |
| Road accessibility (C4) | 1/4 | 1/3 | 1/3 | 1 | 1 | 2 | 1/3 | 0.073 |
| Transport accessibility (C5) | 1/4 | 1/3 | 1/3 | 1 | 1 | 2 | 1/3 | 0.073 |
| Education attainment (C6) | 1/4 | 1/2 | 1/2 | 1/2 | 1/2 | 1 | 1/3 | 0.058 |
| Institutional (C7) | 1 | 2 | 2 | 3 | 3 | 3 | 1 | 0.225 |

Notes: All consistency ratios (CR) < 0.1 (Aspect: 0.0086, Exposure: 0.0067, Sensitivity: 0.0203, Adaptive Capacity: 0.0266).

Supplementary Table S6. AHP matrix of the vulnerability indicators for flood.

| Aspect | A1 | A2 | A3 |  |  |  |  | Weight |
| --- | --- | --- | --- | --- | --- | --- | --- | --- |
| Exposure (A1) | 1 | 3 | 4 |  |  |  |  | 0.614 |
| Sensitivity (A2) | 1/3 | 1 | 1 |  |  |  |  | 0.203 |
| Adaptive Capacity (A3) | 1/4 | 1 | 1 |  |  |  |  | 0.183 |
|  |  |  |  |  |  |  |  |  |
| Exposure | E1 | E2 | E3 |  |  |  |  | Weight |
| Typhoon (E1) | 1 | 2 | 3 |  |  |  |  | 0.526 |
| Flood (E2) | 1/2 | 1 | 2 |  |  |  |  | 0.312 |
| Precipitation (E3) | 1/3 | 1/2 | 1 |  |  |  |  | 0.162 |
|  |  |  |  |  |  |  |  |  |
| Sensitivity | S1 | S2 | S3 | S4 | S5 | S6 |  | Weight |
| Elevation (S1) | 1 | 1 | 2 | 2 | 3 | 4 |  | 0.272 |
| Slope (S2) | 1 | 1 | 3 | 3 | 4 | 5 |  | 0.319 |
| Population density (S3) | 1/2 | 1/3 | 1 | 1 | 1 | 2 |  | 0.107 |
| Urbanization density (S4) | 1/2 | 1/3 | 1 | 1 | 1 | 2 |  | 0.104 |
| Vulnerable population (S5) | 1/3 | 1/4 | 2 | 2 | 1 | 2 |  | 0.137 |
| Gender (S6) | 1/4 | 1/5 | 1/2 | 1/2 | 1/2 | 1 |  | 0.061 |
|  |  |  |  |  |  |  |  |  |
| Adaptive Capacity | C1 | C2 | C3 | C4 | C5 | C6 | C7 | Weight |
| GDP per capita (C1) | 1 | 3 | 2 | 4 | 4 | 4 | 1 | 0.282 |
| Gray Infrastructure^(a)^ (C2) | 1/3 | 1 | 1 | 3 | 3 | 2 | 1/2 | 0.143 |
| Green Infrastructure (C3) | 1/2 | 1 | 1 | 3 | 3 | 2 | 1/2 | 0.145 |
| Road accessibility (C4) | 1/4 | 1/3 | 1/3 | 1 | 1 | 2 | 1/3 | 0.073 |
| Transport accessibility (C5) | 1/4 | 1/3 | 1/3 | 1 | 1 | 2 | 1/3 | 0.073 |
| Education attainment (C6) | 1/4 | 1/2 | 1/2 | 1/2 | 1/2 | 1 | 1/3 | 0.058 |
| Institutional (C7) | 1 | 2 | 2 | 3 | 3 | 3 | 1 | 0.225 |

Notes: All consistency ratios (CR) < 0.1 (Aspect: 0.0086, Exposure: 0.0067, Sensitivity: 0.0265, Adaptive Capacity: 0.0266). (a) Seawall is excluded due to failure to reach consensus from the expert groups (see Supplementary Table 1).

Supplementary Table S7. AHP matrix of the vulnerability indicators for high temperature.

| Aspect | A1 | A2 | A3 |  |  |  |  | Weight |
| --- | --- | --- | --- | --- | --- | --- | --- | --- |
| Exposure (A1) | 1 | 2 | 2 |  |  |  |  | 0.502 |
| Sensitivity (A2) | 1/2 | 1 | 1 |  |  |  |  | 0.250 |
| Adaptive Capacity (A3) | 1/2 | 1 | 1 |  |  |  |  | 0.248 |
|  |  |  |  |  |  |  |  |  |
| Exposure | E1 | E2 |  |  |  |  |  | Weight |
| Precipitation (E1) | 1 | 1/2 |  |  |  |  |  | 0.333 |
| Temperature (E2) | 2 | 1 |  |  |  |  |  | 0.667 |
|  |  |  |  |  |  |  |  |  |
| Sensitivity | S1 | S2 | S3 | S4 |  |  |  | Weight |
| Population density (S1) | 1 | 1 | 1/2 | 3 |  |  |  | 0.246 |
| Urbanization density (S2) | 1 | 1 | 1/3 | 2 |  |  |  | 0.196 |
| Vulnerable population (S3) | 2 | 3 | 1 | 3 |  |  |  | 0.445 |
| Gender (S4) | 1/3 | 1/2 | 1/3 | 1 |  |  |  | 0.113 |
|  |  |  |  |  |  |  |  |  |
| Adaptive Capacity | C1 | C2 | C3 | C4 | C5 | C6 | C7 | Weight |
| GDP per capita (C1) | 1 | 3 | 2 | 4 | 4 | 4 | 1 | 0.275 |
| Gray Infrastructure^(a)^ (C2) | 1/3 | 1 | 1/2 | 2 | 2 | 2 | 1/3 | 0.109 |
| Green Infrastructure^(b)^ (C3) | 1/2 | 2 | 1 | 4 | 3 | 3 | 1/2 | 0.174 |
| Road accessibility (C4) | 1/4 | 1/2 | 1/4 | 1 | 1 | 2 | 1/3 | 0.072 |
| Transport accessibility (C5) | 1/4 | 1/2 | 1/3 | 1 | 1 | 2 | 1/3 | 0.073 |
| Education attainment (C6) | 1/4 | 1/2 | 1/3 | 1/2 | 1/2 | 1 | 1/4 | 0.051 |
| Institutional (C7) | 1 | 3 | 2 | 3 | 3 | 4 | 1 | 0.245 |

Notes: All consistency ratios (CR) < 0.1 (Aspect: 0.0049, Exposure: 0, Sensitivity: 0.0250, Adaptive Capacity: 0.0222). (a) Canal, ditch, and seawall are excluded due to failure to reach consensus from the expert groups; (b) Stormwater drainage system is also excluded by the expert judgement (see Supplementary Table 1).

Supplementary Table S8. AHP matrix of the vulnerability indicators for all hazard together.

| Aspect | A1 | A2 | A3 |  |  |  |  | Weight |
| --- | --- | --- | --- | --- | --- | --- | --- | --- |
| Exposure (A1) | 1 | 3 | 3 |  |  |  |  | 0.607 |
| Sensitivity (A2) | 1/3 | 1 | 1 |  |  |  |  | 0.193 |
| Adaptive Capacity (A3) | 1/3 | 1 | 1 |  |  |  |  | 0.200 |
|  |  |  |  |  |  |  |  |  |
| Exposure | E1 | E2 | E3 | E4 |  |  |  | Weight |
| Typhoon (E1) | 1 | 2 | 3 | 4 |  |  |  | 0.462 |
| Flood (E2) | 1/2 | 1 | 2 | 3 |  |  |  | 0.269 |
| Precipitation (E3) | 1/3 | 1/2 | 1 | 3 |  |  |  | 0.182 |
| Temperature (E4) | 1/4 | 1/3 | 1/3 | 1 |  |  |  | 0.087 |
|  |  |  |  |  |  |  |  |  |
| Sensitivity | S1 | S2 | S3 | S4 | S5 | S6 | S7 | Weight |
| Elevation (S1) | 1 | 1 | 1 | 2 | 2 | 3 | 4 | 0.209 |
| Coastal proximity (S2) | 1 | 1 | 1 | 3 | 3 | 3 | 4 | 0.226 |
| Slope (S3) | 1 | 1 | 1 | 3 | 3 | 4 | 5 | 0.245 |
| Population density (S4) | 1/2 | 1/3 | 1/3 | 1 | 1 | 1/2 | 2 | 0.083 |
| Urbanization density (S5) | 1/2 | 1/3 | 1/3 | 1 | 1 | 1/2 | 2 | 0.081 |
| Vulnerable population (S6) | 1/3 | 1/3 | 1/4 | 2 | 2 | 1 | 3 | 0.109 |
| Gender (S7) | 1/4 | 1/4 | 1/5 | 1/2 | 1/2 | 1/3 | 1 | 0.047 |
|  |  |  |  |  |  |  |  |  |
| Adaptive Capacity | C1 | C2 | C3 | C4 | C5 | C6 | C7 | Weight |
| GDP per capita (C1) | 1 | 3 | 2 | 4 | 4 | 4 | 1 | 0.273 |
| Gray Infrastructure (C2) | 1/3 | 1 | 1 | 3 | 3 | 2 | 1/2 | 0.140 |
| Green Infrastructure (C3) | 1/2 | 1 | 1 | 3 | 3 | 2 | 1/2 | 0.147 |
| Road accessibility (C4) | 1/4 | 1/3 | 1/3 | 1 | 1 | 2 | 1/3 | 0.074 |
| Transport accessibility (C5) | 1/4 | 1/3 | 1/3 | 1 | 1 | 2 | 1/3 | 0.073 |
| Education attainment (C6) | 1/4 | 1/2 | 1/2 | 1/2 | 1/2 | 1 | 1/3 | 0.057 |
| Institutional (C7) | 1 | 2 | 2 | 3 | 3 | 3 | 1 | 0.235 |

Notes: All consistency ratios (CR) < 0.1 (Aspect: 0.0003, Exposure: 0.0401, Sensitivity: 0.0224, Adaptive Capacity: 0.0286). The entries of AHP matrix are average values of the 9 individual expert judgement (2^nd^ round) from government sector, education, and non-government research institutes. All participants have more than 6 years of experience in the fields of flood control, climate disaster mitigation, and/or climate change adaptation. The second round for AHP weighting from the expert groups were conducted from February 6 to 8, 2021.

Supplementary Table S9. Fuzzy AHP matrix of the vulnerability indicators (all hazard).

| Aspect | A1 | A2 | A3 |  |  |  |  |
| --- | --- | --- | --- | --- | --- | --- | --- |
| Exposure (A1) | (1, 1, 1) | (2, 3, 4) | (3, 4, 5) |  |  |  |  |
| Sensitivity (A2) | (, , ) | (1, 1, 1) | (1, 1, 1) |  |  |  |  |
| Adaptive Capacity (A3) | (, , ) | (1, 1, 1) | (1, 1, 1) |  |  |  |  |
|  |  |  |  |  |  |  |  |
| Exposure | E1 | E2 | E3 | E4 |  |  |  |
| Typhoon (E1) | (1, 1, 1) | (1, 1, 1) | (2, 3, 4) | (2, 3, 4) |  |  |  |
| Flood (E2) | (1, 1, 1) | (1, 1, 1) | (1, 2, 3) | (2, 3, 4) |  |  |  |
| Precipitation (E3) | (, , ) | (, , 1) | (1, 1, 1) | (2, 3, 4) |  |  |  |
| Temperature (E4) | (, , ) | (, , ) | (, , ) | (1, 1, 1) |  |  |  |
|  |  |  |  |  |  |  |  |
| Sensitivity | S1 | S2 | S3 | S4 | S5 | S6 | S7 |
| Elevation (S1) | (1, 1, 1) | (1, 1, 1) | (1, 1, 1) | (1, 2, 3) | (1, 2, 3) | (2, 3, 4) | (3, 4, 5) |
| Coastal proximity (S2) | (1, 1, 1) | (1, 1, 1) | (1, 2, 3) | (2, 3, 4) | (2, 3, 4) | (2, 3, 4) | (3, 4, 5) |
| Slope (S3) | (1, 1, 1) | (, , 1) | (1, 1, 1) | (1, 2, 3) | (1, 2, 3) | (3, 4, 5) | (4, 5, 6) |
| Population density (S4) | (, , 1) | (, , ) | (, , 1) | (1, 1, 1) | (1, 1, 1) | (, , 1) | (1, 2, 3) |
| Urbanization density (S5) | (, , 1) | (, , ) | (, , 1) | (1, 1, 1) | (1, 1, 1) | (, , 1) | (1, 2, 3) |
| Vulnerable population (S6) | (, , ) | (, , ) | (, , ) | (1, 2, 3) | (1, 2, 3) | (1, 1, 1) | (1, 2, 3) |
| Gender (S7) | (, , ) | (, , ) | (, , ) | (, , 1) | (, , 1) | (, , 1) | (1, 1, 1) |
|  |  |  |  |  |  |  |  |
| Adaptive Capacity | C1 | C2 | C3 | C4 | C5 | C6 | C7 |
| GDP per capita (C1) | (1, 1, 1) | (2, 3, 4) | (1, 2, 3) | (3, 4, 5) | (3, 4, 5) | (2, 3, 4) | (1, 2, 3) |
| Gray Infrastructure (C2) | (, , ) | (1, 1, 1) | (1, 1, 1) | (2, 3, 4) | (2, 3, 4) | (1, 2, 3) | (1, 1, 1) |
| Green Infrastructure (C3) | (, , 1) | (1, 1, 1) | (1, 1, 1) | (2, 3, 4) | (2, 3, 4) | (1, 2, 3) | (1, 1, 1) |
| Road accessibility (C4) | (, , ) | (, , ) | (, , ) | (1, 1, 1) | (1, 1, 1) | (1, 1, 1) | (, , 1) |
| Transport accessibility (C5) | (, , ) | (, , ) | (, , ) | (1, 1, 1) | (1, 1, 1) | (1, 1, 1) | (, , 1) |
| Education attainment (C6) | (, , ) | (, , 1) | (, , 1) | (1, 1, 1) | (1, 1, 1) | (1, 1, 1) | (, , ) |
| Institutional (C7) | (, , 1) | (1, 1, 1) | (1, 1, 1) | (1, 2, 3) | (1, 2, 3) | (2, 3, 4) | (1, 1, 1) |

Notes: All consistency ratios (CR) < 0.1 (Aspect: 0.0079, Exposure: 0.0442, Sensitivity: 0.0324, Adaptive Capacity: 0.0134).

Supplementary Table S10. Overall weights of the AHP matrix.

| Aspect | Indicator | Typhoon | Flood | Temperature | All Hazard |
| --- | --- | --- | --- | --- | --- |
| Exposure | Typhoon | 0.323 | 0.323 | - | 0.281 |
|  | Flood | 0.191 | 0.191 | - | 0.163 |
|  | Precipitation | 0.100 | 0.100 | 0.167 | 0.110 |
|  | Temperature | - | - | 0.335 | 0.053 |
| Sensitivity | Elevation | 0.044 | 0.055 | - | 0.040 |
|  | Coastal proximity | 0.047 | - | - | 0.044 |
|  | Slope | 0.050 | 0.065 | - | 0.047 |
|  | Population density | 0.017 | 0.022 | 0.062 | 0.016 |
|  | Urbanization density | 0.016 | 0.021 | 0.049 | 0.016 |
|  | Vulnerable population | 0.020 | 0.028 | 0.111 | 0.021 |
|  | Gender | 0.010 | 0.012 | 0.028 | 0.009 |
| Adaptive Capacity | GDP per capita | 0.052 | 0.052 | 0.068 | 0.055 |
|  | Gray Infrastructure | 0.026 | 0.026 | 0.027 | 0.028 |
|  | Green Infrastructure | 0.027 | 0.027 | 0.043 | 0.029 |
|  | Road accessibility | 0.013 | 0.013 | 0.018 | 0.015 |
|  | Transport accessibility | 0.013 | 0.013 | 0.018 | 0.015 |
|  | Education attainment | 0.011 | 0.011 | 0.013 | 0.011 |
|  | Institutional | 0.041 | 0.041 | 0.061 | 0.047 |

Supplementary Table S11. Overall weights of the Fuzzy AHP matrix (all hazard).

| Aspect | Indicator | Local Weight | Global Weight | Δ (AHP-Fuzzy AHP) |
| --- | --- | --- | --- | --- |
| Exposure | Typhoon | 0.370 | 0.233 | 4.73% |
| (0.631) | Flood | 0.334 | 0.211 | -4.75% |
|  | Precipitation | 0.196 | 0.124 | -1.34% |
|  | Temperature | 0.101 | 0.064 | -1.09% |
| Sensitivity | Elevation | 0.203 | 0.039 | 0.09% |
| (0.194) | Coastal proximity | 0.254 | 0.049 | -0.57% |
|  | Slope | 0.206 | 0.040 | 0.73% |
|  | Population density | 0.092 | 0.018 | -0.19% |
|  | Urbanization density | 0.092 | 0.018 | -0.23% |
|  | Vulnerable population | 0.100 | 0.019 | 0.16% |
|  | Gender | 0.053 | 0.010 | -0.11% |
| Adaptive Capacity | GDP per capita | 0.302 | 0.053 | 0.20% |
| (0.175) | Gray Infrastructure | 0.156 | 0.027 | 0.09% |
|  | Green Infrastructure | 0.168 | 0.029 | 0.01% |
|  | Road accessibility | 0.069 | 0.012 | 0.29% |
|  | Transport accessibility | 0.069 | 0.012 | 0.26% |
|  | Education attainment | 0.078 | 0.014 | -0.21% |
|  | Institutional | 0.159 | 0.028 | 1.93% |

Supplementary Table S12. Results of logistic regression by hazard types.

| Hazard | Typhoon | | | Flood | | | Temperature | | |
| --- | --- | --- | --- | --- | --- | --- | --- | --- | --- |
| Period | 2005 – 2010 | 2010 – 2015 | 2015 – 2020 | 2005 – 2010 | 2010 – 2015 | 2015 – 2020 | 2005 – 2010 | 2010 – 2015 | 2015 – 2020 |
| Typhoon | 0.016 | 0.108^**^ | 0.030^***^ | -0.042 | 0.192^***^ | 0.036^***^ | - | - | - |
| Flood | -0.020^*^ | 0.019^***^ | 0.042^***^ | -0.021^**^ | 0.187^***^ | 0.042^***^ | - | - | - |
| Precipitation | 0.064^*^ | 0.040^***^ | 0.071^**^ | 0.057^*^ | 0.021^***^ | 0.101^***^ | -0.031^*^ | 0.188^***^ | 0.073^***^ |
| Temperature | - | - | - | - | - | - | -0.187^**^ | 0.121^***^ | 0.139^***^ |
| Elevation | -0.106^***^ | -0.047^***^ | -0.111^***^ | -0.090^***^ | -0.042^***^ | -0.089^***^ | - | - | - |
| Coastal prox. | 0.021^***^ | -0.045^***^ | -0.007^**^ | - | - | - | - | - | - |
| Slope | 0.068^***^ | 0.029^***^ | 0.044^***^ | 0.050^***^ | 0.023^***^ | 0.034^***^ | - | - | - |
| Pop. density | -0.065^***^ | 0.066^***^ | 0.027 | -0.049^***^ | 0.071^***^ | 0.023^**^ | -0.015^**^ | 0.020^***^ | 0.042^**^ |
| Urban density | -0.643^***^ | 0.025^***^ | 0.034^***^ | -0.050^***^ | 0.022^***^ | 0.025^***^ | -0.243^***^ | 0.088^***^ | 0.168^***^ |
| Vuln. pop. | -0.095^***^ | 0.014 | -0.009 | -0.053^***^ | -0.006 | -0.009 | -0.022^***^ | 0.087 | -0.051 |
| Gender | -0.042 | 0.001 | -0.019 | -0.049 | 0.020 | -0.014 | 0.001 | -0.043 | -0.016 |
| GDP | 0.014 | 0.062^**^ | 0.018^**^ | 0.017^*^ | 0.208^**^ | 0.188^**^ | 0.003 | 0.051^**^ | 0.081^**^ |
| Gray Infra. | 0.042^***^ | -0.038^***^ | 0.009 | 0.040^***^ | -0.033^***^ | -0.01 | 0.043^***^ | 0.034^***^ | -0.027 |
| Green Infra. | 0.021^**^ | -0.012 | 0.105 | 0.025^**^ | -0.020^**^ | 0.092 | 0.019^***^ | -0.102^**^ | -0.040^**^ |
| Road access | 0.076^**^ | -0.063^*^ | -0.016^***^ | 0.050 | -0.112^***^ | -0.016^***^ | 0.218^***^ | -0.115^***^ | -0.027^***^ |
| Public Trans. | -0.011 | -0.148 | -0.030^**^ | -0.003 | -0.026 | -0.028^**^ | 0.025 | -0.018 | -0.015 |
| Education | 0.198^***^ | 0.059^*^ | 0.039^**^ | 0.020^***^ | 0.060^*^ | 0.039^**^ | 0.175^***^ | 0.066^**^ | 0.040 |
| Institutional | -0.012 | 0.050^**^ | 0.041^**^ | -0.011 | 0.054 | 0.043 | -0.008 | 0.029 | 0.039 |
| Constant | 0.876 | -5.448^***^ | -3.360^***^ | 1.831^**^ | -5.223^***^ | -3.313^***^ | 2.431^***^ | -6.429^***^ | -2.170^**^ |
| ROC | 0.901 | 0.852 | 0.870 | 0.901 | 0.851 | 0.869 | 0.897 | 0.853 | 0.855 |
| Pseudo *R*^2^ | 0.365 | 0.256 | 0.326 | 0.363 | 0.252 | 0.326 | 0.354 | 0.254 | 0.304 |

Notes: Observations: 2005-2010 = 2,666,968; 2010-2015 = 2,666,944; and 2015-2020: 1,744,423. Each column reports the coefficient of independent variables. * *p* < 0.10, ** *p* < 0.05, *** *p* < 0.01.

Supplementary Table S13. Experts judgement (typhoon).


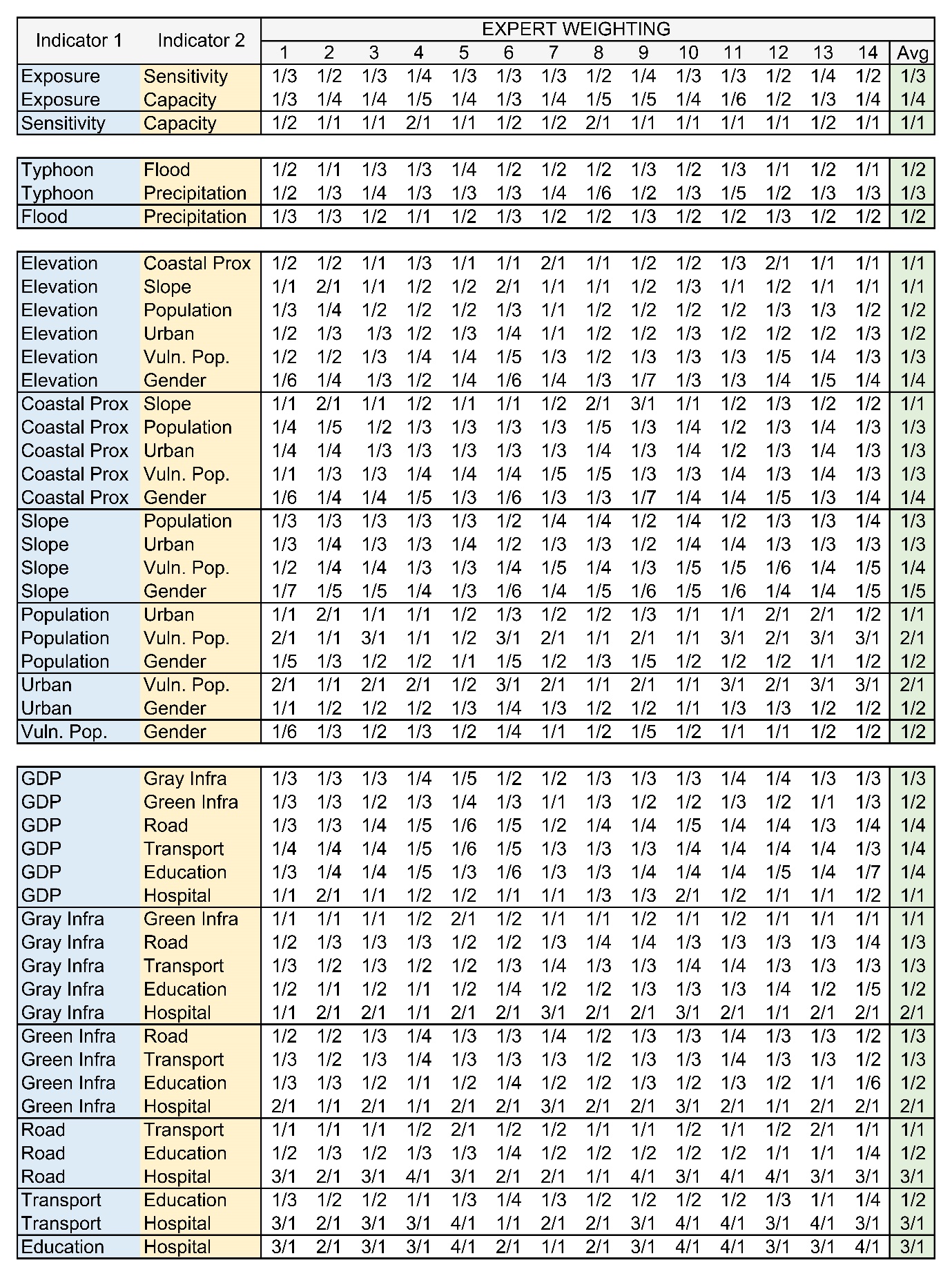


Supplementary Table S14. Experts judgement (flood).


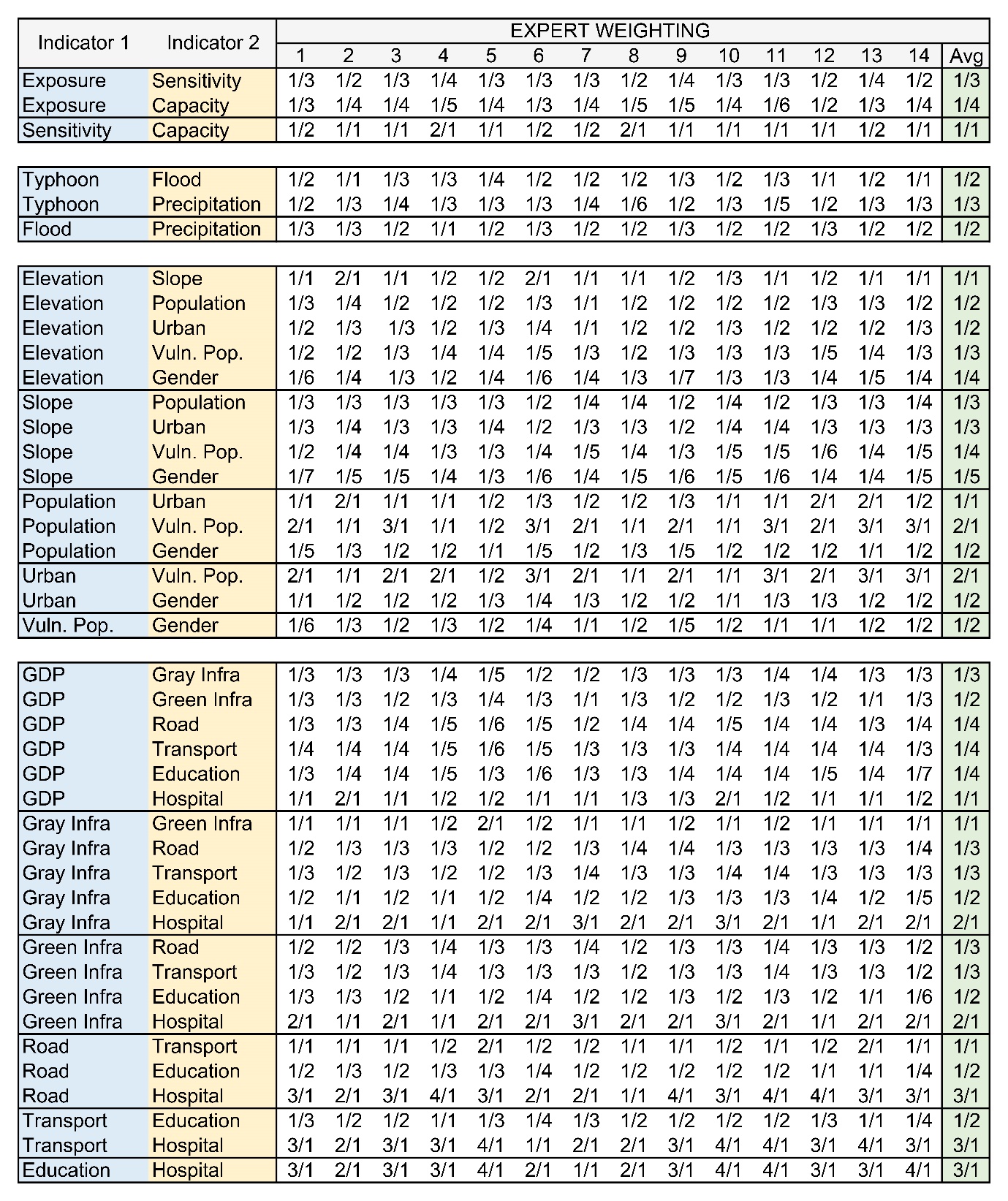


Supplementary Table S15. Experts judgement (high temperature).


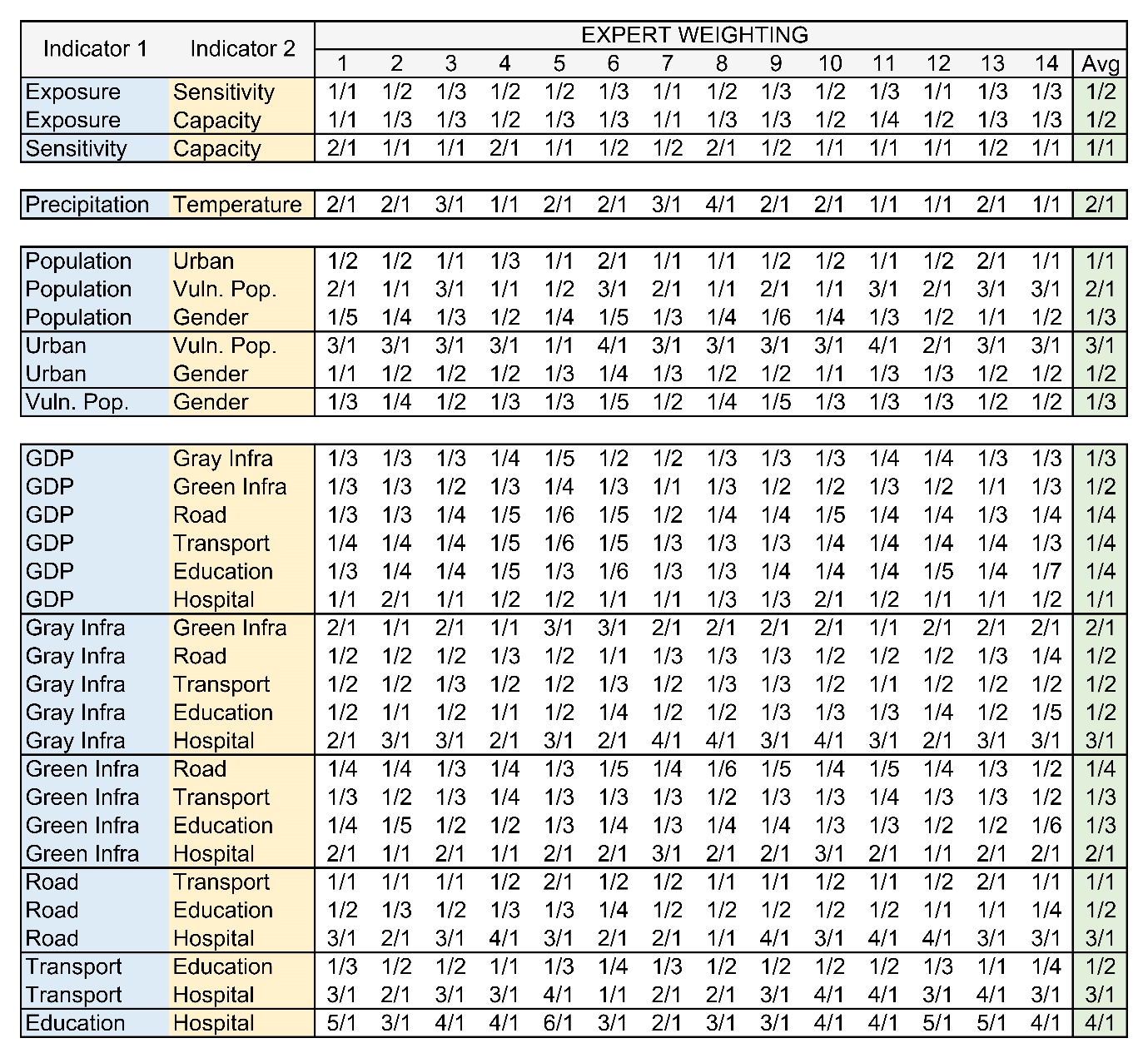


Supplementary Table S16. Experts judgement (all hazard).


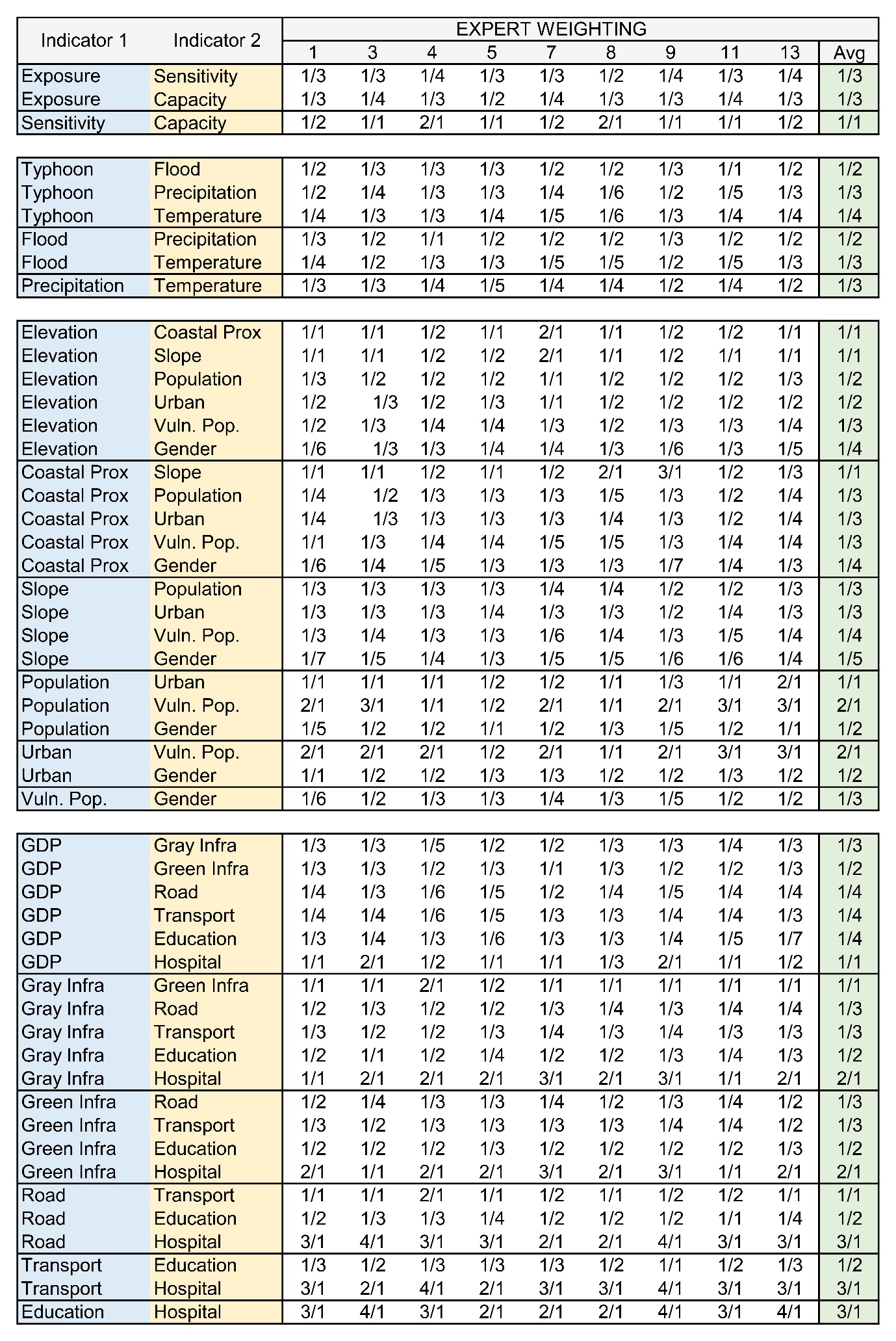


**Supplementary Codes**

Supplementary Code S1. GBA land cover classification (2020)

var table = ee.FeatureCollection("users/finekim/Greater_bay/GBA_bndy0");

var roi = table;

var input = ee.ImageCollection('LANDSAT/LC08/C01/T1')

var image = ee.Algorithms.Landsat.simpleComposite({

collection: input.filterDate('2019-11-01', '2020-02-28'), asFloat: true});

var bands = ['B2', 'B3', 'B4', 'B5', 'B6', 'B7', 'B10', 'B11'];

var polygons = sample2020;

var label = 'landcover';

var training1 = image.addBands(image.select(bands).float()).sample({

region: roi, scale: 30, numPixels: 5000});

var clusterer = ee.Clusterer.wekaKMeans(20).train(training1);

var result = image.addBands(image.select(bands).float()).cluster(clusterer);

var training2 = image.addBands(image.select(bands).float()).sampleRegions({

collection: polygons, properties: [label], scale: 30});

var classifier = ee.Classifier.libsvm({kernelType: 'RBF', gamma: 0.5, cost: 10});

var trained = classifier.train(training2, label, bands);

var classified = result.addBands(image.select(bands).float()).select(bands).classify(trained);

var reducers = ee.Reducer.mean().combine({reducer2: ee.Reducer.stdDev(),

sharedInputs: true});

var maxValue = classified.reduceRegion({reducer: reducers, geometry: roi,

scale: 30, tileScale: 16, maxPixels: 1e9, bestEffort: true});

Map.centerObject(roi, 9);

Map.addLayer(result.clip(roi).randomVisualizer(), {}, 'clusters');

Map.addLayer(image, {bands: ['B4', 'B3', 'B2'], max: 0.5, gamma: 2}); // Landsat 8

Map.addLayer(classified.clip(roi),

{min: 0, max: 6, palette: ['cc0013', '225129',

'cdb33b','91af40','111149','f7e084','aec3d4']}, 'classification');

var stats = ee.Image.pixelArea().addBands(classified)

.reduceRegion({reducer: ee.Reducer.sum().group(1),

geometry: roi, scale: 300});

print(stats);

Export.image.toDrive({image: classified.clip(roi),

description: 'GBA', scale: 30, region: roi, fileFormat: 'GeoTIFF',

maxPixels: 1e9, formatOptions: {cloudOptimized: true}

});

Supplementary Code S2. Accuracy check for the land cover classification

var trainAccuracy = trained.confusionMatrix(); print(trainAccuracy)

var OA = trainAccuracy.accuracy(); var CA = trainAccuracy.consumersAccuracy()

var Kappa = trainAccuracy.kappa(); var Order = trainAccuracy.order()

var PA = trainAccuracy.producersAccuracy()

print(trainAccuracy,'Confusion Matrix'); print(OA,'Overall Accuracy');

print(CA,'Consumers Accuracy'); print(Kappa,'Kappa')

print(Order,'Order'); print(PA,'Producers Accuracy')

var validation = image.addBands(image).sample({numPixels: 5000, seed: 1})

.filter(ee.Filter.neq('B1', null));

var validated = validation.classify(trained);

var testAccuracy = validated.errorMatrix('Land_Cover_Type_1', 'classification');

print('Validation error matrix: ', testAccuracy);

print('Validation overall accuracy: ', testAccuracy.accuracy());

var igbpPalette = ['aec3d4', '152106', '225129', '369b47', '30eb5b', '387242', '6a2325',

'c3aa69', 'b76031', 'd9903d', '91af40', '111149', 'cdb33b', 'cc0013', '33280d',

'd7cdcc', 'f7e084', '6f6f6f'];

Map.centerObject(roi, 9);

Map.addLayer(image.clip(roi), {bands: ['B3', 'B2', 'B1'], max: 0.4}, 'landsat');

Map.addLayer(classified.clip(roi), {palette: igbpPalette, min: 0, max: 17}, 'classification');

var sample = image.addBands(image).sample({numPixels: 5000, seed: 0});

sample = sample.randomColumn();

var split = 0.7; // 70% training, 30% testing.

var training = sample.filter(ee.Filter.lt('random', split));

var validation = sample.filter(ee.Filter.gte('random', split));

var sample = image.addBands(image).sample({

region: landsat.geometry(), numPixels: 5000, seed: 0, geometries: true, tileScale: 16});

sample = sample.randomColumn();

var split = 0.7; // 70% training, 30% testing.

var training = sample.filter(ee.Filter.lt('random', split)); print(training.size());

var validation = sample.filter(ee.Filter.gte('random', split));

var distFilter = ee.Filter.withinDistance({distance: 1000,

leftField: '.geo', rightField: '.geo', maxError: 10});

var join = ee.Join.inverted(); training = join.apply(training, validation, distFilter); print(training.size());

Supplementary Code S3. Identification of flood risk area (2015-2020)

var startyear = 2015; var endyear = 2020;

var roi = table;

var startdate = ee.Date.fromYMD(startyear,1,1);

var enddate = ee.Date.fromYMD(endyear,12,31)

var DIFF_THRESHOLD = 0.5;

var cloud_thresh = 40;

var ndwi_viz = {min:-1.0, max:1.0, palette:"a3a3a3,e9ff34,53db46,6699CC"};

var l5images = l5.filterDate(startdate,enddate).filterBounds(roi);

var l7images = l7.filterDate(startdate,enddate).filterBounds(roi);

var l8images = l8.filterDate(startdate,enddate).filterBounds(roi);

var cloudfunction = function(image){

var CloudScore = ee.Algorithms.Landsat.simpleCloudScore(image);

var quality = CloudScore.select('cloud'); var cloud01 = quality.gt(cloud_thresh);

var cloudmask = image.mask().and(cloud01.not()); return image.updateMask(cloudmask);};

l5images = l5images.map(cloudfunction); l7images = l7images.map(cloudfunction);

l8images = l8images.map(cloudfunction);

l5images = l5images.select(["B2","B4"]); l7images = l7images.select(["B2","B4"]);

l8images = l8images.select(["B3","B5"]);

function l8Ndwi(img) {var ndwi = img.normalizedDifference(['B3', 'B5']).rename('NDWI');

return img.addBands(ndwi);}

function l57Ndwi(img) {var ndwi = img.normalizedDifference(['B2', 'B4']).rename('NDWI');

return img.addBands(ndwi);}

var l5ndwi = l5images.map(l57Ndwi); var l7ndwi = l7images.map(l57Ndwi);

var l8ndwi = l8images.map(l8Ndwi);

var allcollection = ee.ImageCollection((l5ndwi.merge(l7ndwi)).merge(l8ndwi));

print(allcollection)

var dry = allcollection.select("NDWI").reduce(ee.Reducer.percentile([10]))

var wet = allcollection.select("NDWI").reduce(ee.Reducer.percentile([90]));

dry = dry.gt(0.3); wet = wet.gt(0.0)

var diff = wet.subtract(dry)

var indundated = diff.gt(DIFF_THRESHOLD)

Map.centerObject(roi, 8);

Map.addLayer(dry.clip(roi),ndwi_viz,"dry"); Map.addLayer(wet.clip(roi),ndwi_viz, "wet");

Map.addLayer(diff.clip(roi),ndwi_viz, "diff");

Map.addLayer(indundated.mask(indundated).clip(roi),{palette:"0000FF"}, "inundated");

Supplementary Code S4. Elevation

var roi = table;

var dataset = ee.Image('USGS/SRTMGL1_003');

var elevation = dataset.select('elevation').clip(roi);

var elevationVis = {min: -100.0, max: 10000.0, palette: ['FF5733', 'EBFD07']};

Map.centerObject(roi, 9);

Map.addLayer(elevation.clip(roi), elevationVis, 'Elevation');

Supplementary Code S5. Slope

var roi = table;

var dataset = ee.Image('USGS/SRTMGL1_003');

var slope = ee.Terrain.slope(dataset);

Map.centerObject(roi, 9);

Map.addLayer(slope.clip(roi), {min: 0, max: 60}, 'slope');

Supplementary Code S6. Night-time light index (2015-2019)

var roi = table;

var VIIRS = ee.ImageCollection('NOAA/VIIRS/DNB/MONTHLY_V1/VCMCFG');

var night15_19 = VIIRS

.filterDate('2015-01-01', '2019-12-31').mosaic().clip(roi).select('avg_rad');

var night_VIIRS = {min: 0.0, max: 60.0};

Map.centerObject(roi, 8);

Map.addLayer(night15_19, night_VIIRS, 'Night 2015-2019');
